# Supplementary material for: Azathioprine immunosuppression and disease modification in Parkinson’s disease (AZA-PD): a randomised double-blind placebo-controlled phase II trial protocol
Source: BMJ Open. 2020 Nov 23;10(11):e040527. doi: 10.1136/bmjopen-2020-040527 (PMC7684836; doi:10.1136/bmjopen-2020-040527)
Supplement: Author's manuscript [file bmjopen-2020-040527.draft_revisions.pdf]

BMJ Open is committed to open peer review. As part of this commitment we make the peer review history of every article we publish publicly available.

When an article is published we post the peer reviewers' comments and the authors' responses online. We also post the versions of the paper that were used during peer review. These are the versions that the peer review comments apply to.

The versions of the paper that follow are the versions that were submitted during the peer review process. They are not the versions of record or the final published versions. They should not be cited or distributed as the published version of this manuscript.

BMJ Open is an open access journal and the full, final, typeset and author-corrected version of record of the manuscript is available on our site with no access controls, subscription charges or pay-per-view fees (http://bmjopen.bmj.com).

If you have any questions on BMJ Open's open peer review process please email info.bmjopen@bmj.com

BMJ Open

Azathioprine immunosuppression and disease modification in Parkinson's disease (AZA-PD): a randomised double-blind placebo-controlled phase II trial protocol

Journal:	*BMJ Open*
Manuscript ID	bmjopen-2020-040527
Article Type:	Protocol
Date Submitted by the Author:	15-May-2020
Complete List of Authors:	Greenland, Julia; University of Cambridge, Department of Clinical Neurosciences<br>Cutting, Emma; University of Cambridge, Department of Clinical Neurosciences; Cambridge Clinical Trials Unit<br>Kadyan, Sonakshi; Cambridge Clinical Trials Unit<br>Bond, Simon; Cambridge Clinical Trials Unit<br>Chhabra, Anita; Cambridge University Hospitals NHS Foundation Trust, Pharmacy<br>Williams-Gray, Caroline; University of Cambridge, Department of Clinical Neurosciences
Keywords:	Parkinson-s disease < NEUROLOGY, THERAPEUTICS, IMMUNOLOGY

SCHOLARONE™ Manuscripts

I, the Submitting Author has the right to grant and does grant on behalf of all authors of the Work (as defined in the below author licence), an exclusive licence and/or a non-exclusive licence for contributions from authors who are: i) UK Crown employees; ii) where BMJ has agreed a CC-BY licence shall apply, and/or iii) in accordance with the terms applicable for US Federal Government officers or employees acting as part of their official duties; on a worldwide, perpetual, irrevocable, royalty-free basis to BMJ Publishing Group Ltd ("BMJ") its licensees and where the relevant Journal is co-owned by BMJ to the co-owners of the Journal, to publish the Work in this journal and any other BMJ products and to exploit all rights, as set out in our licence.

The Submitting Author accepts and understands that any supply made under these terms is made by BMJ to the Submitting Author unless you are acting as an employee on behalf of your employer or a postgraduate student of an affiliated institution which is paying any applicable article publishing charge ("APC") for Open Access articles. Where the Submitting Author wishes to make the Work available on an Open Access basis (and intends to pay the relevant APC), the terms of reuse of such Open Access shall be governed by a Creative Commons licence – details of these licences and which Creative Commons licence will apply to this Work are set out in our licence referred to above.

Other than as permitted in any relevant BMJ Author's Self Archiving Policies, I confirm this Work has not been accepted for publication elsewhere, is not being considered for publication elsewhere and does not duplicate material already published. I confirm all authors consent to publication of this Work and authorise the granting of this licence.

For peer review only

Title

Azathioprine immunosuppression and disease modification in Parkinson's disease (AZA-PD): a randomised double-blind placebo-controlled phase II trial protocol

Authors

Julia C. Greenland[1], Emma Cutting[1,2], Sonakshi Kadyan[2], Simon Bond[2], Anita Chhabra[3], Caroline H. Williams-Gray[1]

[1.]Department of Clinical Neurosciences, John van Geest Centre for Brain Repair, University of Cambridge, Cambridge, UK.

[2]Cambridge Clinical Trials Unit, Cambridge University Hospitals NHS Foundation Trust, Cambridge

[3] Pharmacy, Cambridge University Hospitals NHS Foundation Trust, Cambridge

Corresponding author

Julia Greenland

John van Geest Centre for Brain Repair, Department of Clinical Neurosciences, University of Cambridge, Forvie Site, Cambridge CB2 0PY, United Kingdom.

Tel: +44 1223 331160

Fax: +44 1223 331174

Jcg69@cam.ac.uk

Keywords

Parkinson's disease, immune system, immunosuppression, clinical trial, therapy

Word Count

3951

For peer review only

For peer review only

Abstract

Introduction

The immune system is implicated in the aetiology and progression of Parkinson's disease (PD). Inflammation and immune activation occur both in the brain and in the periphery, and a pro-inflammatory cytokine profile is associated with more rapid clinical progression. Furthermore, the risk of developing PD is related to genetic variation in immune-related genes, and reduced by the use of immunosuppressant medication. We are therefore conducting a 'proof of concept' trial of azathioprine, an immunosuppressant medication, to investigate whether suppressing the peripheral immune system has a disease-modifying effect in PD.

Methods and analysis

AZA-PD is a phase II randomised placebo-controlled double-blind trial in early PD. Sixty participants, with clinical markers indicating an elevated risk of disease progression and no inflammatory or immune comorbidity, will be treated (azathioprine: placebo, 1:1) for 12 months, with a further 6-months follow-up. The primary outcome is the change in the MDS-UPDRS gait/axial score in the OFF state over the 12-month treatment period. Exploratory outcomes include additional measures of motor and cognitive function, non-motor symptoms and quality of life. In addition, peripheral and central immune markers will be investigated through analysis of blood, cerebrospinal fluid and PK-11195 PET imaging.

Ethics and dissemination

The study was approved by the London-Westminster research ethics committee (reference 19/LO/1705) and has been accepted by the MHRA for a clinical trials authorisation (reference CTA 12854/0248/001-0001). In addition, approval has been granted from the Administration of Radioactive Substances Advisory Committee (ARSAC). The results of this trial will be disseminated

For peer review only

through publication in scientific journals and presentation at national and international conferences and a lay summary will be available on our website.

Trial Registration

ISRCTN14616801, 14/5/2020. EudraCT- 2018-003089-14

Protocol version 1.1: AZA-PD CCTU0218

Strengths and limitations of this study

- First clinical trial of a peripherally acting immunosuppressive drug in Parkinson's disease
- Robust, randomised double-blind placebo-controlled design
- Novel patient stratification approach with recruitment of a more rapidly progressing subgroup to optimise chance of demonstrating clinical effect
- Detailed exploratory measures examining peripheral and central immune profile in PD to demonstrate proof of mechanism
- As a single centre 'proof of concept' trial, sample size is limited to 60 participants.

For peer review only

INTRODUCTION

Parkinson's disease (PD) is a common neurodegenerative disorder diagnosed clinically by key motor features. The core pathology in PD involves the loss of dopaminergic neurons in the substantia nigra (SN) with intracellular accumulation of alpha-synuclein aggregates (Lewy bodies). Dopamine replacement therapy can control some of the motor symptoms. However, other problems including impaired balance and cognitive dysfunction are due to more widespread neurodegenerative pathology and are consequently unresponsive to dopaminergic therapies. These symptoms progress such that by 10 years from diagnosis, around two thirds of patients have balance and walking difficulties, and around half have dementia,[1] with a profound impact on quality of life [2, 3] and care requirements.[4] There are currently no treatments to alter disease course and prevent these devastating complications, hence there is an urgent need to find effective disease-modifying therapies for PD. There is increasing evidence that the immune system plays an important role in driving neurodegeneration in PD, and we propose that targeting the immune system may be an effective strategy for slowing disease progression.

The link between genetic variation in immune pathways and risk of Parkinson's disease is well-established. Risk of developing PD is associated with polymorphisms in the Human Leucocyte Antigen (HLA) region, which encodes proteins vital to antigen recognition and presentation.[5, 6] Large scale analyses of Genome Wide Association Study (GWAS) data also implicate the immune system in PD, demonstrating heritability enrichment for genes of the adaptive immune system, including those involved in lymphocyte regulation and cytokine signalling pathways.[7, 8] Further evidence of an immune contribution to PD risk comes from epidemiological studies: individuals who regularly take non-steroidal anti-inflammatory drugs (NSAIDs) have reduced risk of developing PD [9, 10], as do those on immunosuppressive therapy.[11] There is also evidence that immune activation impacts on disease progression rate. In a large incident PD cohort, a pro-inflammatory serum cytokine profile at baseline

For peer review only

was associated with faster motor progression and impaired cognition over 36 months of follow-up.[12]

Activation of microglia, the inflammatory cells of the brain, has been clearly demonstrated in PD patients both at post-mortem [13-16] and using [^{11}C]-PK11195 positron emission tomography (PET) imaging in-vivo.[17-19] These cells have a role in responding to tissue injury, regulating the cerebral microenvironment, and antigen presentation.[20] It is thought that this activation is driven by toxic misfolded or post-translationally modified forms of alpha-synuclein released by degenerating cells, leading to secretion of proinflammatory and neurotoxic molecules, resulting in a cyclical process of cell damage.[21]

Abnormalities in the peripheral immune profile in PD are also well demonstrated and include alterations in both the innate and adaptive immune compartments. There is a shift towards 'classical' (inflammatory) monocytes with elevated expression of activation markers,[22] particularly in patients at higher dementia risk.[23] In the T lymphocyte compartment, several authors have reported bias towards pro-inflammatory CD4+ lymphocyte subsets and production of pro-inflammatory cytokines.[24-27] There may also be a reduction in the number and function of CD4+ T-regulatory (Treg) cells, whose role is to counter this pro-inflammatory response.[24, 26] In addition, changes in the CD8 compartment have been reported, with increased expression of activation markers and reduced markers of age-related senescence.[28]

Importantly, T-cells with specificity for epitopes of alpha-synuclein have been identified and reported to occur at higher frequency in PD than controls; furthermore their frequency was closely associated with possession of known PD risk alleles at the HLA locus[29], thus suggesting that alpha-synuclein may drive a peripheral adaptive immune response as well as an innate response of microglia in the brain. Elevated levels of alpha-synuclein specific antibodies are also present in the early stages of PD.[30] Peripheral immune cells may contribute to brain inflammation and neurotoxicity by trafficking into the central nervous system in PD. CD4+ and CD8+ lymphocytes have been shown to be present

For peer review only

in increased numbers in the SN at post-mortem in PD patients,[16, 25] as well as in ex-vivo cerebrospinal fluid (CSF) samples.[31] The precise mechanism by which peripheral immune cells drive neuronal damage in PD is still unclear, but it has recently been demonstrated that Th17 cells from PD patients drive cell death in autologous iPSC-derived dopaminergic midbrain neurons.[25]

Immune manipulation in animal models of PD alters disease susceptibility and severity. Using an MPTP mouse model of PD, studies have demonstrated that a lack of CD4+ lymphocytes attenuates dopaminergic cell death,[16] as does administration of Treg cells.[32] In mice that over-express alpha-synuclein, knockout of MHCII prevents both microglial activation and dopaminergic neurodegeneration.[33] Furthermore, cyclosporin, a widely used immunosuppressant, is effective in improving motor and cognitive deficits in multiple mouse models of PD.[34]

Although animal models of PD indicate that immunomodulatory therapies may have efficacy in protecting against neurodegeneration, there is limited clinical trial data in PD to date. Phase II trials of minocycline and pioglitazone, agents which reduce microglial activation in the brain in animal models, have been negative.[35-37] An early phase trial of sargramostim, a human recombinant granulocyte-macrophage colony-stimulating factor which promotes differentiation of pro-inflammatory T-effector cells into Treg cells has reported a modest improvement in an exploratory outcome of motor function over 8-weeks treatment.[38]

We propose that direct suppression of the peripheral immune system is an alternative, highly relevant therapeutic strategy which has not been tested in clinical trials to date. Azathioprine is an immunosuppressant drug widely used in clinical practice for a range of immune-related conditions. It is a purine analogue which inhibits nucleic acid synthesis, hence reducing proliferation of lymphocytes involved in targeting and amplification of the immune response. It affects both the cell-mediated and antibody-mediated responses through reducing T and B lymphocyte proliferation.[39] It was selected over other immunosuppressants because of its established efficacy in a range of clinical conditions, including central nervous system disorders such as multiple sclerosis,[40] and its acceptable safety

For peer review only

profile with recognised protocols for toxicity monitoring. Furthermore, it is generally well tolerated in the elderly and is a once-daily preparation for ease of administration.

METHODS AND ANALYSIS

Overview

AZA-PD is a randomised double-blind placebo-controlled trial of azathioprine in early Parkinson's disease which aims to provide 'proof of concept' that a peripherally acting immunosuppressive drug can slow clinical disease progression. The trial will investigate whether the drug has an effect on disease course over 12 months of treatment and whether this is maintained over 6 months of subsequent follow-up. Sixty participants will be recruited and randomised 1:1 to receive active treatment or placebo. Clinical assessments will be performed at baseline, 6 months, 12 months and 18 months (6 months post completion of treatment), with rigorous safety monitoring. In addition, the trial aims to demonstrate 'proof of mechanism' by evaluating the impact of azathioprine on blood, CSF and neuroimaging parameters of immune activation in the trial population and determining the relationship between these parameters and clinical measures of disease progression.

The trial timeline is summarised in **Error! Reference source not found.**.

Although AZA-PD is open to recruitment, given the current COVID-19 pandemic, recruitment has not commenced due to safety concerns. The Trial Steering Committee (TSC) and Data and Safety Monitoring Board (DSMB) will decide on an appropriate date to begin recruitment in due course, and protocol amendments to maximise patient safety will be submitted to the appropriate bodies when the best course of action has been determined.

For peer review only

Patient and public involvement (PPI)

PD patients and their partners and carers who attend our research clinic at the John van Geest Centre for Brain Repair (VGB), University of Cambridge gave input into the protocol design. A PPI advisory panel of 4 patients/carers reviewed the protocol and provided specific feedback, leading to the addition of optional components. The PPI panel also reviewed our participant information sheet for clarity.

Participant Identification

Participants will be recruited from a single site in Cambridge, UK. Potential participants will be identified from the PD Research clinic database at the VGB. These individuals have undergone detailed clinical phenotyping, and information on demographics, comorbidities and medication is available. They have consented to be contacted about other research studies. Potential participants will be pre-selected by cross-referencing existing data with the inclusion/exclusion criteria outlined in **Error! Reference source not found.**. A key component of this process involves the calculation of a prognostic risk score, based on a model we have previously developed and validated, using age, MDS-UPDRS axial score and semantic fluency to estimate risk of a poor prognosis (dementia, postural instability or death) within 5 years.[41] Only patients with a risk greater than 50% will be invited to take part. This strategy has been adopted to maximise the probability of demonstrating significant slowing of clinical progression with azathioprine treatment.

Potential participants will be sent a copy of the participant information sheet, and telephoned after two weeks to determine whether they are interested in participating. If so, they will attend a screening visit, where written informed consent will be obtained before confirming eligibility.

For peer review only

Eligibility criteria

A potential participant will be deemed eligible for recruitment into AZA-PD if they meet the inclusion and exclusion criteria listed in **Error! Reference source not found.**. A review of medical history and blood tests will be used to determine eligibility.

Outcome measures

The primary outcome measure is change in MDS-UPDRS gait/axial score in the OFF state over the 12-month treatment period. This is a clinical measure which has been shown to be the most sensitive measure of motor progression in PD, is relatively resistant to dopaminergic therapy and has an important impact on quality of life.[42] This score is a sum of the points from the following sections of MDS-UPDRS part III:

- 3.1 – speech
- 3.2 – facial expression
- 3.9 – rising from a chair
- 3.10 – gait
- 3.12 – postural stability
- 3.13 – posture
- 3.14 – body bradykinesia

Other outcome measures are exploratory and include:

- change in MDS-UPDRS gait/axial score in OFF state at 18 months
- change in total MDS-UPDRS in OFF state at 12 and 18 months
- change in electromagnetic sensor (EMS) measurements whilst performing MDS-UPDRS tremor and bradykinesia assessments at 12 and 18 months

For peer review only

- proportion of patients developing postural instability (Hoehn and Yahr stage 3 or greater) at 12 and 18 months
- change in global cognition (ACE-III) at 12 and 18 months
- change in patient reported outcome measure of quality of life (PDQ-39) at 12 and 18 months
- change in NMSS at 12 and 18 months
- change in dose of symptomatic dopaminergic therapy (LEDD) at 12 and 18 months
- the safety and tolerability of azathioprine assessed by the number of adverse events (AEs) recorded during the 12-month treatment period
- change in [^{11}C]-PK11195 PET non- dissociable binding potential (BP_{ND}) in subcortical and cortical regions of interest at 12 months
- change in total lymphocyte count at 6, 12 and 18 months
- change in serum immunoglobulin levels at 6, 12 and 18 months
- change in levels of serum and CSF cytokine levels at 12 and 18 months
- change in lymphocyte subsets in blood and CSF at 12 and 18 months

Sample Size Calculation

The treatment effect size is unknown and therefore cannot be used to inform sample size calculations. A sample size of 60 has been selected pragmatically based on feasibility of recruitment.

However, longitudinal clinical data from the ICICLE-PD cohort study provides some idea of an anticipated effect size for the primary outcome measure. ICICLE-PD patients were stratified by cytokine profile. Those with a 'pro-inflammatory' profile (n=32) had a more rapid symptom progression, with mean (SD) annualised change in MDS-UPDRS gait/axial score of 1.95 (1.92). In the subgroup with an 'anti-inflammatory' cytokine profile (n=26), mean (SD) annualised change in MDS-

For peer review only

UPDRS gait/axial score was 0.72 (1.40).[12] The corresponding between group difference of 1.2 points is equivalent to a standardised effect size (Cohen's d) of 0.73. The magnitude this effect, which equates to a 4% change on the 28-point gait-axial MDS-UPDRS subscale, would be clinically significant. For comparison, the estimated minimum clinically important change on the full 132-point MDS-UPDRS motor scale is ≈2% (2.5 points).[43] Furthermore, the axial-gait items of the MDS-UPDRS are those with the greatest impact on quality of life.[42]

As this is an early-phase proof of concept trial, it is important to maximise the chances of continuing development if the treatment is genuinely effective, and thus a significance level of 25% under a 1-sided test will be used. If the treatment effect is a 2% change (0.37 standardised effect), the design has 78% power, and for a 4% change (0.73 standardised effect), the design has 99% power.

Trial procedures

Clinical

Clinical measures assessing both motor and non-motor components of PD will be performed at baseline, mid-treatment, end-of-treatment and after 6-months further follow-up (see **Error! Reference source not found.**). Throughout the course of the trial participants will continue to take their PD medication as prescribed by their treating physician, and dose adjustments are permitted. However, some assessments will be conducted 'OFF' medication.

The Movement Disorder Society-Unified Parkinson's Disease Rating Scale (MDS-UPDRS) is widely used to quantify PD severity and includes questionnaires assessing the non-motor and motor aspects of the disease, a motor examination performed by a clinician and an assessment of motor complications (dyskinesias and fluctuations).[44] The MDS-UPDRS part III will be assessed in the OFF state; in the absence of regular dopaminergic medication. The aim of this is to expose underlying disease severity and avoid confounding effects from variability in medication doses or timing. This

For peer review only

examination will be filmed to enable subsequent rating by an independent assessor to check inter-rater reliability. Our primary outcome measure is derived from the MDS-UPDRS: the gait-axial sub-score, as previously discussed. Two sections of the MDS-UPDRS part III (tremor and bradykinesia) will be repeated whilst the participant is wearing an electromagnetic sensor (EMS, Polhemus Inc.) on the index finger and thumb, which will give an objective measure of the participant's movements. Motor stage will also be evaluated using the Hoehn and Yahr scale, a 5-point scale used to capture the stages of progression of PD, with stage 3 representing the development of postural instability.[45]

Cognition will be assessed using the Addenbrooke's Cognitive Examination-III (ACE-III). This provides a global measure of cognition as well as sub-scores in 5 domains; attention, memory, fluency, language and visuospatial function.[46] Other non-motor aspects of PD will be evaluated using the short form 15-item Geriatric Depression Scale (GDS), a questionnaire assessing depressive symptoms filled in by the participant,[47] and the Parkinson's disease Non-Motor Symptom Scale (NMSS), completed by the trial assessor.[48] Finally, we will use the Parkinson's Disease Questionnaire 39 (PDQ-39), a self-rated questionnaire measuring PD-related quality of life.[49]

Dopaminergic medication requirement will be monitored throughout the trial, and standardised by calculating Levodopa Equivalent Daily Dose (LEDD), which allows quantification of different doses and types of Parkinson's medication on a single scale.[50]

PK-11195 PET imaging

[^{11}C]-PK11195 PET will be used to measure activated microglia in the brain.[17, 18] Scanning will be conducted at the Wolfson Brain Imaging Centre (WBIC) on a GE SIGNA PET/MRI scanner, with the radiotracer produced at the WBIC Radiopharmaceutical Chemistry laboratory. MRI will be used for co-localisation. 500 MBq of the[^{11}C]-PK11195 radiotracer will be injected via a peripheral venous

For peer review only

cannula over 30 seconds and PET emission data will be acquired for 75 min post-injection in 55 time frames. Following image reconstruction and attenuation correction, specific tracer binding will be analysed with the simplified reference tissue model (SRTM; [51]) to quantify binding potential relative to a non-displaceable compartment (BPND). The reference region will be estimated with supervised cluster analysis for [^{11}C]PK11195 from existing scans in healthy controls acquired on the same scanner. BPND will be compared pre and post treatment using a region of interest approach.

Given that some participants may have difficulty tolerating prolonged PET imaging, this will be optional. It will be performed between screening and baseline, and repeated within 3 months following the end of treatment.

Biosample collection and processing

14 mls of blood will be collected in serum tubes at baseline, mid-treatment, end-of-treatment and follow-up visits for analysis of inflammatory cytokines, CRP and immunoglobulins. Tubes will be centrifuged at 2000RPM (600G) for 15 mins following 15 minutes clotting time for extraction of serum. Aliquots will be stored at -80°C for subsequent batch analysis using ELISA and electrochemiluminescence assays.

At baseline, end-of-treatment and follow-up visits an additional 27mls of blood will be collected in lithium heparin tubes for separation of peripheral blood mononuclear cells (PBMCs) for immunophenotyping. A concurrent full blood count (FBC) will be performed from an EDTA sample (2.6mls).

CSF will be collected via lumbar puncture before the baseline visit and at the treatment endpoint. This is an optional component of the study in order to ensure that its inclusion does not limit recruitment. CSF will be spun at 400G for 10 minutes for extraction of immune cells for contemporaneous immunophenotyping alongside PBMC analysis. Supernatants will be stored at -80°C for later batch analysis of relevant immune and protein markers. Immunophenotyping will be

For peer review only

performed for subsets of T cells, B cells and monocytes using flow cytometry, run within 24 hours of sample collection.

For peer review only

Table 1: Schedule of Assessments

		**Screening Visit**<br>Day -42 (max)	**Imaging Visit**<br>Approx. Day -14±	**CSF Collection**<br>Approx. Day -7±	**Baseline Visit**<br>Day 0± 14	**Monitoring Visits[1]**<br>Day 14 ± 5 and onwards	**Dose escalation Visit**<br>Day 28± 5	**Mid-Treatment Visit**<br>Day 182± 14	**End of Treatment Visit**<br>Day 365± 14	**Imaging Visit**<br>Day 410± 45	**Follow-up Visit**<br>Day 547±14
	Informed written consent	✓									
	Eligibility review	✓									
	Randomisation				✓						
	Vital Signs				✓	✓	✓	✓	✓		✓
	Weight in kg	✓			✓	✓	✓	✓			
Clinical Assessments	Demographics				✓						
	Medical history	✓			✓	✓	✓	✓	✓		✓
	Concomitant medication review	✓			✓	✓	✓	✓	✓		✓
	MDS-UPDRS				✓			✓	✓		✓
	NART				✓						
	ACE III				✓			✓	✓		✓
	GDS				✓			✓	✓		✓
	NMSS				✓			✓	✓		✓
	PDQ-39				✓			✓	✓		✓
	Adverse events review	✓	✓	✓	✓	✓	✓	✓	✓	✓	✓
	IMP compliance check					✓	✓	✓	✓		

For peer review only

Imaging-Optional	[11C]-PK11195 PET-MRI		✓							✓	
Blood tests	Screening bloods[2]	✓									
	Safety monitoring bloods[3]					✓	✓	✓	✓		✓
	CRP, immunoglobulins and serum storage for cytokine measurement			✓				✓	✓		✓
	Immunophenotyping			✓					✓		✓
CSF-optional	Immune markers and immunophenotyping			✓					✓		

[1] Monitoring visits will take place at: day 14, day 42, day 56, day 70, day 98, day 252 and day 547 (as part of the routine follow-up visit). Additional monitoring visits may also be scheduled if there are patient safety concerns.

[2]Screening bloods include FBC, U&Es, LFTs, coagulation, TPMT, HIV, Syphilis, HepB, HepC, EBV serology, VZV serology, LH and FSH (if female and reproductive age)

[3]Monitoring bloods include FBC, U&Es, LFTs

For peer review only

Treatment allocation, blinding and safety monitoring

Participants will be randomised 1:1 to receive azathioprine or placebo using Sealed Envelope, an online randomisation system. Clinical assessors and participants will be blinded to treatment allocation. Balanced assignment of each treatment will be achieved using permuted block randomisation, which will be stratified for: age ≤ 71 vs > 71, and MDS-UPDRS-III ≤ 30 vs > 30.

Treatment will be commenced at a dose of 1mg/kg, based on 25mg tablets of IMP (azathioprine/placebo). In addition to the visits shown in *Figure 1*, treatment monitoring visits will be conducted to screen for potential complications associated with azathioprine. These will include blood tests to screen for myelosuppression, liver or renal dysfunction, adverse events reporting and assessment of treatment compliance (review of patient-completed dosing diary and counting of IMP at regular intervals). Initially, monitoring visits will occur 2 weekly, and after 4 weeks, the azathioprine dose will be increased to 2mg/kg (assuming blood tests and clinical assessments are satisfactory), the standard therapeutic dose used in clinical practice. There will be a matched doubling of the placebo dose to maintain blinding. Once the participant is stable on their dose, treatment monitoring will be carried out less frequently (see monitoring protocol, *Figure 3*).

Given that azathioprine will produce changes in FBC parameters, the blinded trial team conducting patient assessments and laboratory analysis will not have access to monitoring blood results throughout the duration of the trial. The blood tests will be reviewed by a separate unblinded team of clinicians, who will make decisions on dose changes when necessary. If a dose reduction is required, the participant will have an additional 3 monitoring visits at 2-weekly intervals to ensure stability of blood tests. Dose reductions and, where necessary, withdrawal of treatment will be carried out based on pre-defined clinical and laboratory criteria to ensure the safety of participants, including the development of significant myelosuppression, intolerable gastrointestinal side effects and hypersensitivity reactions. Participants who have been withdrawn from treatment will be encouraged to continue to attend the remainder of the trial assessments.

To ensure blinding is maintained amongst clinical assessors and participants, dose adjustments and treatment withdrawals will be also made for some participants in the placebo arm, with additional monitoring visits. Matched pairs of placebo and azathioprine-treated participants will be generated to facilitate this, and all dose adjustment decisions will be made by the unblinded team.

Emergency unblinding will be carried out in the event of a valid medical or safety reason, where the clinical care of the participant will be facilitated by the knowledge of whether they have been taking azathioprine, as decided by the treating clinician. It will be executed using Sealed Envelope, and where possible the trial team will remain blinded.

Following the end of the trial, and for participants who withdraw early, we will offer continuing follow-up through our research clinic at the VGB.

Trial monitoring and oversight

Safety monitoring will be overseen by a DSMB who will have access to interim recruitment and safety data. The DSMB will report to the TSC should it become clear that one treatment allocation is either indicated or contraindicated, or apparent that no clear outcome can be obtained from the trial. The TSC, who are independent from the Sponsor, will provide overall supervision of the trial and ensure that it meets appropriate standards. These groups include clinicians with experience in PD or immunosuppression, independent statisticians, and the TSC includes a lay member.

AZA-PD is jointly sponsored by Cambridge University Hospitals NHS Foundation Trust and the University of Cambridge. The Sponsor will review all trial documentation, including any proposed amendments, prior to submission to the relevant regulatory bodies, which can only be completed once the Sponsor has approved the changes. Changes will then be communicated to participants, the DMSB, TSC, and trial registries.

For peer review only

Adherence to the protocol and regulatory requirements will be reviewed by a Clinical Trials Monitor, assigned by the Sponsor. The first monitoring visit will occur within 10 days of the first randomisation, with frequency thereafter determined by a risk assessment which will be reviewed and adjusted as necessary throughout the course of the trial.

Data Analysis

Trial data will be transferred from paper CRFs to the electronic trial database, where it will be anonymised, but with preserved linkage records. Patient-identifiable data (PID), will be stored on a password-protected database within the SDHS hosted by the University of Cambridge, with access granted only to relevant members of the trial team. PID will be kept for 5 years following the end of the trial, as per regulatory requirements. Participant consent will be specifically sought for data/sample sharing with our collaborators, and use of remaining biological samples in future ethically-approved research.

Data will be analysed on an "intention to treat" basis, with further "per protocol" analysis in participants with at least 80% compliance with trial medication. All endpoints will be summarised and broken down by treatment group and time point, where relevant. Mean, median, standard deviation, minimum/maximum will be used for continuous endpoints, and frequency tables for categorical or binary endpoints. Equivalent box and whisker plots or stacked bar charts will be produced for continuous and categorical endpoints respectively.

The primary analysis will estimate the difference between treatment groups in terms of the primary endpoint. An analysis of covariance (ANCOVA) model will be fitted adjusting for baseline MDS-UPDRS gait/axial score, gender, LEDD, and age. Treatment effect estimates, standard errors, confidence intervals (95% and 40% levels) and 1-sided p-values will be provided. A 1-sided p-value less than 25% will be regarded as statistically significant. Similar comparative analyses will be produced for other

For peer review only

time points of the MDS-UPDRS gait/axial score and exploratory endpoints, using ANCOVA for continuous endpoints or logistic regression for categorical or binary endpoints.

Longitudinal data will use a mixed effect model repeated measurements (MMRM) analysis, to include an unstructured patient-level random effect for nominal visit, visit and visit-treatment interaction fixed effects at visits post-baseline, with adjustment for baseline covariates. To assess the slope of change over time, the longitudinal data will be analysed using a similar MMRM but with a fixed effect of time from randomisation as a continuous, rather than nominal covariate, with a treatment-time interaction to compare treatment groups and patient-level random effect for slope, with adjustment for baseline covariates.

ETHICS AND DISSEMINATION

This study was approved by the London-Westminster research ethics committee (reference 19/LO/1705) and has been accepted by the MHRA for a clinical trials authorisation (reference CTA 12854/0248/001-0001). In addition, approval has been granted from the Administration of radioactive substances advisory committee (ARSAC).

We will feedback trial results to participants and our wider cohort of research participants via our annual PD Open Day and newsletter. A lay summary of the results will be available on our website. The results will also be disseminated through publication in scientific journals and presentation at national and international conferences.

AZA-PD has been accepted onto the NIHR Clinical Research Network (CRN) portfolio and details of this trial are also available on the following registries: ISRCTN14616801, EudraCT- 2018-003089-14.

For peer review only

REFERENCES

1. Williams-Gray CH, Mason SL, Evans JR, et al. The CamPaIGN study of Parkinson's disease: 10-year outlook in an incident population-based cohort. *Journal of neurology, neurosurgery, and psychiatry* 2013;84(11):1258-64. doi: 10.1136/jnnp-2013-305277 [published Online First: 2013/06/20]
2. Schrag A, Jahanshahi M, Quinn N. What contributes to quality of life in patients with Parkinson's disease? *Journal of neurology, neurosurgery, and psychiatry* 2000;69(3):308-12. [published Online First: 2000/08/17]
3. Evans JR, Mason SL, Williams-Gray CH, et al. The natural history of treated Parkinson's disease in an incident, community based cohort. *Journal of neurology, neurosurgery, and psychiatry* 2011;82(10):1112-8. doi: 10.1136/jnnp.2011.240366 [published Online First: 2011/05/20]
4. Aarsland D, Larsen JP, Tandberg E, et al. Predictors of nursing home placement in Parkinson's disease: a population-based, prospective study. *Journal of the American Geriatrics Society* 2000;48(8):938-42. [published Online First: 2000/09/01]
5. Saiki M, Baker A, Williams-Gray CH, et al. Association of the human leucocyte antigen region with susceptibility to Parkinson's disease. *Journal of neurology, neurosurgery, and psychiatry* 2010;81(8):890-1. doi: 10.1136/jnnp.2008.162883 [published Online First: 2010/05/14]
6. International Parkinson Disease Genomics Consortium, Nalls MA, Plagnol V, et al. Imputation of sequence variants for identification of genetic risks for Parkinson's disease: a meta-analysis of genome-wide association studies. *Lancet* 2011;377(9766):641-9.
7. Gagliano SA, Pouget JG, Hardy J, et al. Genomics implicates adaptive and innate immunity in Alzheimer's and Parkinson's diseases. *Annals of clinical and translational neurology* 2016;3(12):924-33. doi: 10.1002/acn3.369 [published Online First: 2017/01/18]

For peer review only

8. Holmans P, Moskvina V, Jones L, et al. A pathway-based analysis provides additional support for an immune-related genetic susceptibility to Parkinson's disease. *Human molecular genetics* 2013;22(5):1039-49. doi: 10.1093/hmg/dds492 [published Online First: 2012/12/12]
9. Gao X, Chen H, Schwarzschild MA, et al. Use of ibuprofen and risk of Parkinson disease. *Neurology* 2011;76(10):863-9. doi: 10.1212/WNL.0b013e31820f2d79 [published Online First: 2011/03/04]
10. Chen H, Zhang SM, Hernan MA, et al. Nonsteroidal anti-inflammatory drugs and the risk of Parkinson disease. *Archives of neurology* 2003;60(8):1059-64. doi: 10.1001/archneur.60.8.1059 [published Online First: 2003/08/20]
11. Racette BA, Gross A, Vouri SM, et al. Immunosuppressants and risk of Parkinson disease. *Annals of clinical and translational neurology* 2018;5(7):870-75. doi: 10.1002/acn3.580 [published Online First: 2018/07/17]
12. Williams-Gray CH, Wijeyekoon R, Yarnall AJ, et al. Serum immune markers and disease progression in an incident Parkinson's disease cohort (ICICLE-PD). *Movement disorders : official journal of the Movement Disorder Society* 2016;31(7):995-1003. doi: 10.1002/mds.26563
13. McGeer PL, Itagaki S, Boyes BE, et al. Reactive microglia are positive for HLA-DR in the substantia nigra of Parkinson's and Alzheimer's disease brains. *Neurology* 1988;38(8):1285-91. [published Online First: 1988/08/01]
14. Mogi M, Harada M, Kondo T, et al. Interleukin-1 beta, interleukin-6, epidermal growth factor and transforming growth factor-alpha are elevated in the brain from parkinsonian patients. *Neuroscience letters* 1994;180(2):147-50. [published Online First: 1994/10/24]
15. Loeffler DA, Camp DM, Conant SB. Complement activation in the Parkinson's disease substantia nigra: an immunocytochemical study.

For peer review only

Journal of neuroinflammation 2006;3:29. doi: 10.1186/1742-2094-3-29 [published Online First: 2006/10/21]

16. Brochard V, Combadiere B, Prigent A, et al. Infiltration of CD4+ lymphocytes into the brain contributes to neurodegeneration in a mouse model of Parkinson disease. *The Journal of clinical investigation* 2009;119(1):182-92. doi: 10.1172/jci36470 [published Online First: 2008/12/24]
17. Gerhard A, Pavese N, Hotton G, et al. In vivo imaging of microglial activation with [11C](R)-PK11195 PET in idiopathic Parkinson's disease. *Neurobiology of disease* 2006;21(2):404-12. doi: 10.1016/j.nbd.2005.08.002 [published Online First: 2005/09/27]
18. Ouchi Y, Yoshikawa E, Sekine Y, et al. Microglial activation and dopamine terminal loss in early Parkinson's disease. *Annals of neurology* 2005;57(2):168-75. doi: 10.1002/ana.20338 [published Online First: 2005/01/26]
19. Edison P, Ahmed I, Fan Z, et al. Microglia, amyloid, and glucose metabolism in Parkinson's disease with and without dementia. *Neuropsychopharmacology : official publication of the American College of Neuropsychopharmacology* 2013;38(6):938-49. doi: 10.1038/npp.2012.255 [published Online First: 2013/01/11]
20. Perry VH. Innate inflammation in Parkinson's disease. *Cold Spring Harbor perspectives in medicine* 2012;2(9):a009373. doi: 10.1101/cshperspect.a009373 [published Online First: 2012/09/07]
21. Gao HM, Hong JS. Why neurodegenerative diseases are progressive: uncontrolled inflammation drives disease progression. *Trends in immunology* 2008;29(8):357-65. doi: 10.1016/j.it.2008.05.002 [published Online First: 2008/07/05]
22. Grozdanov V, Bliederhaeuser C, Ruf WP, et al. Inflammatory dysregulation of blood monocytes in Parkinson's disease patients. *Acta neuropathologica* 2014;128(5):651-63. doi: 10.1007/s00401-014-1345-4

For peer review only

23. Wijeyekoon RS, Kronenberg-Versteeg D, Scott KM, et al. Peripheral innate immune and bacterial signals relate to clinical heterogeneity in Parkinson's disease. *Brain, behavior, and immunity* 2020 doi: 10.1016/j.bbi.2020.01.018 [published Online First: 2020/02/02]
24. Kustrimovic N, Comi C, Magistrelli L, et al. Parkinson's disease patients have a complex phenotypic and functional Th1 bias: cross-sectional studies of CD4+ Th1/Th2/T17 and Treg in drug-naive and drug-treated patients. *Journal of neuroinflammation* 2018;15(1):205. doi: 10.1186/s12974-018-1248-8 [published Online First: 2018/07/14]
25. Sommer A, Maxreiter F, Krach F, et al. Th17 Lymphocytes Induce Neuronal Cell Death in a Human iPSC-Based Model of Parkinson's Disease. *Cell stem cell* 2018;23(1):123-31.e6. doi: 10.1016/j.stem.2018.06.015 [published Online First: 2018/07/07]
26. Saunders JA, Estes KA, Kosloski LM, et al. CD4+ regulatory and effector/memory T cell subsets profile motor dysfunction in Parkinson's disease. *Journal of neuroimmune pharmacology : the official journal of the Society on NeuroImmune Pharmacology* 2012;7(4):927-38. doi: 10.1007/s11481-012-9402-z
27. Baba Y, Kuroiwa A, Uitti RJ, et al. Alterations of T-lymphocyte populations in Parkinson disease. *Parkinsonism & related disorders* 2005;11(8):493-8. doi: 10.1016/j.parkreldis.2005.07.005 [published Online First: 2005/09/13]
28. Williams-Gray CH, Wijeyekoon RS, Scott KM, et al. Abnormalities of age-related T cell senescence in Parkinson’s disease. *Journal of Neuroinflammation* 2018;15:166. doi: 10.1186/s12974-018-1206-5
29. Sulzer D, Alcalay RN, Garretti F, et al. T cells from patients with Parkinson's disease recognize alpha-synuclein peptides. *Nature* 2017;546(7660):656-61. doi: 10.1038/nature22815 [published Online First: 2017/06/22]

For peer review only

30. Scott KM, Kouli A, Yeoh SL, et al. A Systematic Review and Meta-Analysis of Alpha Synuclein Auto-Antibodies in Parkinson's Disease. *Frontiers in neurology* 2018;9:815. doi: 10.3389/fneur.2018.00815 [published Online First: 2018/10/20]
31. Schroder JB, Pawlowski M, Meyer Zu Horste G, et al. Immune Cell Activation in the Cerebrospinal Fluid of Patients With Parkinson's Disease. *Frontiers in neurology* 2018;9:1081. doi: 10.3389/fneur.2018.01081 [published Online First: 2019/01/09]
32. Reynolds AD, Stone DK, Hutter JA, et al. Regulatory T cells attenuate Th17 cell-mediated nigrostriatal dopaminergic neurodegeneration in a model of Parkinson's disease. *Journal of immunology (Baltimore, Md : 1950)* 2010;184(5):2261-71. doi: 10.4049/jimmunol.0901852 [published Online First: 2010/02/02]
33. Harms AS, Cao S, Rowse AL, et al. MHCII is required for alpha-synuclein-induced activation of microglia, CD4 T cell proliferation, and dopaminergic neurodegeneration. *The Journal of neuroscience : the official journal of the Society for Neuroscience* 2013;33(23):9592-600. doi: 10.1523/jneurosci.5610-12.2013 [published Online First: 2013/06/07]
34. Tamburrino A, Churchill MJ, Wan OW, et al. Cyclosporin promotes neurorestoration and cell replacement therapy in pre-clinical models of Parkinson's disease. *Acta neuropathologica communications* 2015;3:84. doi: 10.1186/s40478-015-0263-6 [published Online First: 2015/12/17]
35. Investigators NN-P. A pilot clinical trial of creatine and minocycline in early Parkinson disease: 18-month results. *Clin Neuropharmacol* 2008;31(3):141-50. doi: https://dx.doi.org/10.1097/WNF.0b013e3181342f32
36. NINDS Net-Pd Investigators. A randomized, double-blind, futility clinical trial of creatine and minocycline in early Parkinson disease. *Neurology* 2006;66(5):664-71. doi: 10.1212/01.wnl.0000201252.57661.e1

For peer review only

37. Investigators NETiPDF-Z. Pioglitazone in early Parkinson's disease: a phase 2, multicentre, double-blind, randomised trial.[Erratum appears in Lancet Neurol. 2015 Oct; 14(10):979]. *Lancet neurol* 2015;14(8):795-803. doi: https://dx.doi.org/10.1016/S1474-4422(15)00144-1

38. Gendelman HE, Zhang Y, Santamaria P, et al. Evaluation of the safety and immunomodulatory effects of sargramostim in a randomized, double-blind phase 1 clinical Parkinson's disease trial. *NPJ Parkinson's disease* 2017;3:10. doi: 10.1038/s41531-017-0013-5 [published Online First: 2017/06/27]

39. Lennard L. The clinical pharmacology of 6-mercaptopurine. *European journal of clinical pharmacology* 1992;43(4):329-39. doi: 10.1007/bf02220605 [published Online First: 1992/01/01]

40. Casetta I, Iuliano G, Filippini G. Azathioprine for multiple sclerosis. *The Cochrane database of systematic reviews* 2007(4):Cd003982. doi: 10.1002/14651858.CD003982.pub2 [published Online First: 2007/10/19]

41. Velseboer DC, de Bie RM, Wieske L, et al. Development and external validation of a prognostic model in newly diagnosed Parkinson disease. *Neurology* 2016;86(11):986-93. doi: 10.1212/wnl.0000000000002437 [published Online First: 2016/02/19]

42. Evans JR, Mason SL, Williams-Gray CH, et al. The factor structure of the UPDRS as an index of disease progression in Parkinson's disease. *Journal of Parkinson's disease* 2011;1(1):75-82. doi: 10.3233/jpd-2011-0002 [published Online First: 2011/01/01]

43. Shulman LM, Gruber-Baldini AL, Anderson KE, et al. The clinically important difference on the unified Parkinson's disease rating scale. *Archives of neurology* 2010;67(1):64-70. doi: 10.1001/archneurol.2009.295

44. Goetz CG, Tilley BC, Shaftman SR, et al. Movement Disorder Society-sponsored revision of the Unified Parkinson's Disease Rating Scale

For peer review only

(MDS-UPDRS): scale presentation and clinimetric testing results. *Movement disorders : official journal of the Movement Disorder Society* 2008;23(15):2129-70. doi: 10.1002/mds.22340

45. Hoehn MM, Yahr MD. Parkinsonism: onset, progression and mortality. *Neurology* 1967;17(5):427-42. [published Online First: 1967/05/01]
46. Hsieh S, Schubert S, Hoon C, et al. Validation of the Addenbrooke's Cognitive Examination III in frontotemporal dementia and Alzheimer's disease. *Dementia and geriatric cognitive disorders* 2013;36(3-4):242-50. doi: 10.1159/000351671 [published Online First: 2013/08/21]
47. Sheikh JI YJ. Geriatric Depression Scale (GDS): recent evidence and development of a shorter version. *Clin Gerontol* 1986;5(June):165-73.
48. Chaudhuri KR, Martinez-Martin P, Brown RG, et al. The metric properties of a novel non-motor symptoms scale for Parkinson's disease: Results from an international pilot study. *Movement disorders : official journal of the Movement Disorder Society* 2007;22(13):1901-11. doi: 10.1002/mds.21596
49. Jenkinson C, Fitzpatrick R, Peto V, et al. The Parkinson's Disease Questionnaire (PDQ-39): development and validation of a Parkinson's disease summary index score. *Age and ageing* 1997;26(5):353-7. [published Online First: 1997/11/14]
50. Tomlinson CL, Stowe R, Patel S, et al. Systematic review of levodopa dose equivalency reporting in Parkinson's disease. *Movement disorders : official journal of the Movement Disorder Society* 2010;25(15):2649-53. doi: 10.1002/mds.23429 [published Online First: 2010/11/12]
51. Lammertsma AA, Hume SP. Simplified reference tissue model for PET receptor studies. *NeuroImage* 1996;4(3 Pt 1):153-8. doi: 10.1006/nimg.1996.0066 [published Online First: 1996/12/01]

For peer review only

52. Dubois, B., et al., *Diagnostic procedures for Parkinson's disease dementia: recommendations from the movement disorder society task force.* Mov Disord, 2007. **22**(16): p. 2314-24.

Acknowledgements

This research was supported by the National Institute for Health Research (NIHR) Cambridge Biomedical Research Centre and the Cambridge Clinical Trials Unit (CCTU).

For peer review only

Authors' Contributions

CWG is the CI of this trial. JG is sub-investigator of this trial.

CWG and JG – study design and writing the protocol.

SK, EC and AC – critical review of the protocol.

SB - statistical analysis plan.

Sponsorship

This study is jointly sponsored by the University of Cambridge and Cambridge University Hospitals NHS foundation Trust.

Contact:

Ms Carrie Bayliss

Cambridge Clinical Trials Unit

Cambridge University Hospitals NHSFT

Hills Road

Cambridge

CB2 0QQ

E-mail: cctu@addenbrookes.nhs.uk

Funding Statement

This trial is funded by Cambridge Centre for Parkinson-Plus and the Cure Parkinson's Trust (grant ref CW011), and supported by the NIHR Cambridge Biomedical Research Centre (grant ref no 146281). CHWG is supported by a RCUK/UKRI Research Innovation Fellowship awarded by the Medical Research Council (MR/R007446/1).

The funders have no role in study design; collection, management, analysis, and interpretation of data; writing of the report; or the decision to submit the report for publication.

Competing Interests Statement

None declared

For peer review only

TABLE OF FIGURES

Figure 1: Overview of trial timeline

Figure 2: AZA-PD eligibility criteria

Figure 3: Treatment monitoring schedule

For peer review only

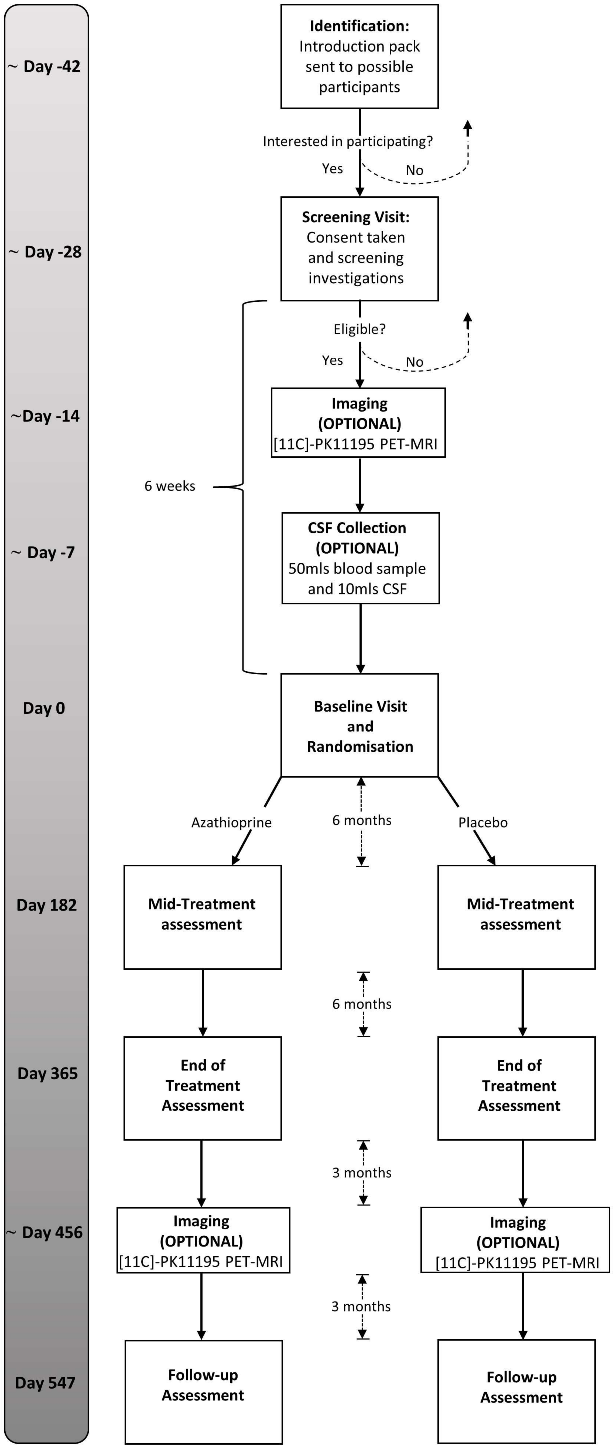
~ Day -42
~ Day -28
~Day -14
~ Day -7
Day 0
Day 182
Day 365
~ Day 456
Day 547
Identification: Introduction pack sent to possible participants
Interested in participating?
Yes
No
Screening Visit: Consent taken and screening investigations
Eligible?
Yes
No
Imaging (OPTIONAL) [11C]-PK11195 PET-MRI
6 weeks
CSF Collection (OPTIONAL) 50mls blood sample and 10mls CSF
Baseline Visit and Randomisation
Azathioprine
6 months
Placebo
Mid-Treatment assessment
Mid-Treatment assessment
6 months
End of Treatment Assessment
End of Treatment Assessment
3 months
Imaging (OPTIONAL) [11C]-PK11195 PET-MRI
Imaging (OPTIONAL) [11C]-PK11195 PET-MRI
3 months
Follow-up Assessment
Follow-up Assessment

Inclusion Criteria

- be capable of giving signed informed consent
- be aged over 50 years
- be a fluent English speaker
- have a diagnosis of PD according to UKPDS Brain Bank Criteria
- have a disease duration of less than 3 years
- have a probability of poor outcome (postural instability/dementia/death) at 5 years from diagnosis ≥50% [41]
- have adequate organ and marrow function, as defined below (measured within 42 days of first dose of trial medication):
    - Haemoglobin ≥ 110 g/L
    - Platelet count ≥ 130 x 10^9/L
    - Neutrophil count ≥ 1.5 x 10^9/L
    - Renal function- creatinine clearance ≥50mL/min.
    - Hepatic function- ALT and bilirubin ≤2 times the institutional upper limit of normal

Exclusion Criteria

- The use of prescribed immunomodulatory or regular anti-inflammatory drugs
- Known inflammatory or autoimmune disease, or chronic or latent infection
- Active infection requiring the use of parenteral antimicrobial agents within 2 months prior to the first dose of trial treatment
- Skin or solid organ malignancy within the 5 years prior to the screening assessment
- The inability to take or swallow oral medication
- Parkinson's Disease Dementia according to MDS PD Dementia criteria [52]
- A positive test for HIV or Hepatitis B or C
- TPMT deficiency
- A lack of immunity to VZV
- Negative EBV IgG
- Chronic liver disease
- Renal impairment - creatinine clearance <50mL/min
- Current or previous haematological malignancy
- Concomitant allopurinol
- Any concurrent medical or psychiatric condition or disease that is likely to interfere with the trial procedures or results, or that in the opinion of the investigator, would constitute a hazard for participating in this trial
- Receipt of live, attenuated vaccine within the 30 days prior to the screening assessment
- Women of childbearing potential. Female patients must be surgically sterile or be postmenopausal
- Male patients must be surgically sterile or must agree to use effective contraception during the period of therapy and for 6 months after the last dose of the trial treatment
- Known hypersensitivity to azathioprine or its excipient
- Received an investigational drug or used an invasive investigational medical device within 4 weeks before the screening assessment or is currently enrolled in an interventional investigational trial

For peer review only - http://bmjopen.bmj.com/site/about/guidelines.xhtml

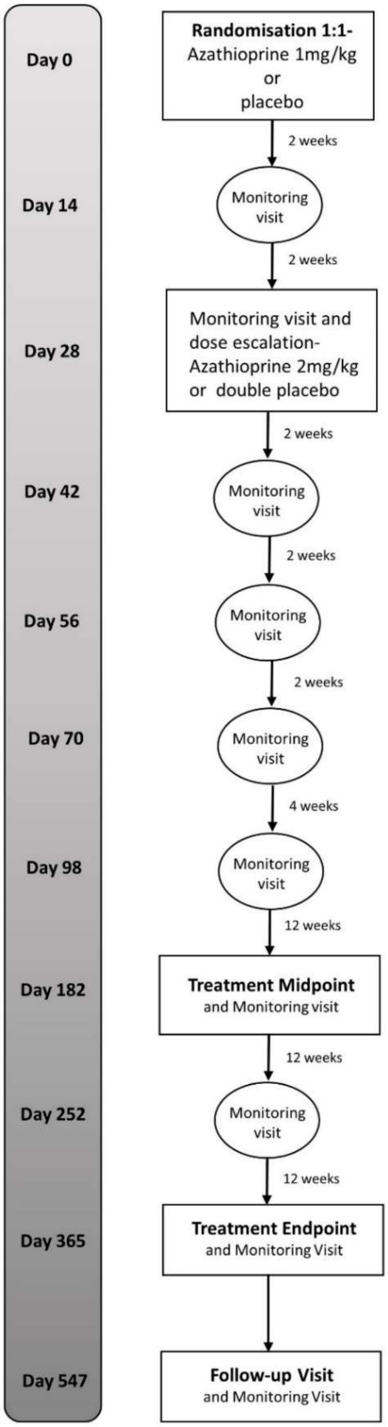
Day 0
Randomisation 1:1-
Azathioprine 1mg/kg
or
placebo
2 weeks
Day 14
Monitoring visit
2 weeks
Day 28
Monitoring visit and dose escalation-
Azathioprine 2mg/kg
or double placebo
2 weeks
Day 42
Monitoring visit
2 weeks
Day 56
Monitoring visit
2 weeks
Day 70
Monitoring visit
4 weeks
Day 98
Monitoring visit
12 weeks
Day 182
Treatment Midpoint
and Monitoring visit
12 weeks
Day 252
Monitoring visit
12 weeks
Day 365
Treatment Endpoint
and Monitoring Visit
Day 547
Follow-up Visit
and Monitoring Visit

STANDARD PROTOCOL ITEMS: RECOMMENDATIONS FOR INTERVENTIONAL TRIALS

SPIRIT 2013 Checklist: Recommended items to address in a clinical trial protocol and related documents*

Section/item	Item No	Description
**Administrative information**		
Title	1	Descriptive title identifying the study design, population, interventions, and, if applicable, trial acronym ✓
Trial registration	2a	Trial identifier and registry name. If not yet registered, name of intended registry ✓
	2b	All items from the World Health Organization Trial Registration Data Set ✓
Protocol version	3	Date and version identifier ✓
Funding	4	Sources and types of financial, material, and other support "funding statement" ✓
Roles and responsibilities	5a	Names, affiliations, and roles of protocol contributors "Authors' contribution" ✓
	5b	Name and contact information for the trial sponsor "Sponsor contact"✓
	5c	Role of study sponsor and funders, if any, in study design; collection, management, analysis, and interpretation of data; writing of the report; and the decision to submit the report for publication, including whether they will have ultimate authority over any of these activities "Trial monitoring and oversight" and "Funding statement"✓
	5d	Composition, roles, and responsibilities of the coordinating centre, steering committee, endpoint adjudication committee, data management team, and other individuals or groups overseeing the trial, if applicable (see Item 21a for data monitoring committee) – safety monitoring and authors' contributions✓
**Introduction**		
Background and rationale	6a	Description of research question and justification for undertaking the trial, including summary of relevant studies (published and unpublished) examining benefits and harms for each intervention – covered in the introduction ✓

For peer review only

	6b	Explanation for choice of comparators – see last paragraph of introduction ✓
Objectives	7	Specific objectives or hypotheses – in “trial overview” ✓
Trial design	8	Description of trial design including type of trial (eg, parallel group, crossover, factorial, single group), allocation ratio, and framework (eg, superiority, equivalence, noninferiority, exploratory) – “trial overview” ✓

Methods: Participants, interventions, and outcomes

Study setting	9	Description of study settings (eg, community clinic, academic hospital) and list of countries where data will be collected. Reference to where list of study sites can be obtained – “Participant identification” ✓
Eligibility criteria	10	Inclusion and exclusion criteria for participants. If applicable, eligibility criteria for study centres and individuals who will perform the interventions (eg, surgeons, psychotherapists) “Inclusion criteria” and “exclusion criteria”. ✓
Interventions	11a	Interventions for each group with sufficient detail to allow replication, including how and when they will be administered – “trial procedures” ✓
	11b	Criteria for discontinuing or modifying allocated interventions for a given trial participant (eg, drug dose change in response to harms, participant request, or improving/worsening disease) “Treatment allocation and safety monitoring” ✓
	11c	Strategies to improve adherence to intervention protocols, and any procedures for monitoring adherence (eg, drug tablet return, laboratory tests) – “Treatment allocation and safety monitoring” ✓
	11d	Relevant concomitant care and interventions that are permitted or prohibited during the trial See “overview” and exclusion criteria. ✓
Outcomes	12	Primary, secondary, and other outcomes, including the specific measurement variable (eg, systolic blood pressure), analysis metric (eg, change from baseline, final value, time to event), method of aggregation (eg, median, proportion), and time point for each outcome. Explanation of the clinical relevance of chosen efficacy and harm outcomes is strongly recommended –“outcome measures” ✓
Participant timeline	13	Time schedule of enrolment, interventions (including any run-ins and washouts), assessments, and visits for participants. A schematic diagram is highly recommended (see Figure) Figure 1 and table 1 ✓
Sample size	14	Estimated number of participants needed to achieve study objectives and how it was determined, including clinical and statistical assumptions supporting any sample size calculations “sample size calculation” ✓

Recruitment	15	Strategies for achieving adequate participant enrolment to reach target sample size "participant identification" ✓

Methods: Assignment of interventions (for controlled trials)

Allocation:

Sequence generation	16a	Method of generating the allocation sequence (eg, computer-generated random numbers), and list of any factors for stratification. To reduce predictability of a random sequence, details of any planned restriction (eg, blocking) should be provided in a separate document that is unavailable to those who enrol participants or assign interventions IMP, treatment allocation and safety monitoring ✓
Allocation concealment mechanism	16b	Mechanism of implementing the allocation sequence (eg, central telephone; sequentially numbered, opaque, sealed envelopes), describing any steps to conceal the sequence until interventions are assigned Treatment allocation and safety monitoring ✓
Implementation	16c	Who will generate the allocation sequence, who will enrol participants, and who will assign participants to interventions Treatment allocation and safety monitoring ✓
Blinding (masking)	17a	Who will be blinded after assignment to interventions (eg, trial participants, care providers, outcome assessors, data analysts), and how Treatment allocation and safety monitoring ✓
	17b	If blinded, circumstances under which unblinding is permissible, and procedure for revealing a participant's allocated intervention during the trial Treatment allocation and safety monitoring ✓

Methods: Data collection, management, and analysis

Data collection methods	18a	Plans for assessment and collection of outcome, baseline, and other trial data, including any related processes to promote data quality (eg, duplicate measurements, training of assessors) and a description of study instruments (eg, questionnaires, laboratory tests) along with their reliability and validity, if known. Reference to where data collection forms can be found, if not in the protocol "trial procedures" ✓
	18b	Plans to promote participant retention and complete follow-up, including list of any outcome data to be collected for participants who discontinue or deviate from intervention protocols Treatment allocation and safety monitoring ✓
Data management	19	Plans for data entry, coding, security, and storage, including any related processes to promote data quality (eg, double data entry; range checks for data values). Reference to where details of data management procedures can be found, if not in the protocol Data analysis ✓

Statistical methods	20a	Statistical methods for analysing primary and secondary outcomes. Reference to where other details of the statistical analysis plan can be found, if not in the protocol – Data analysis ✓
	20b	Methods for any additional analyses (eg, subgroup and adjusted analyses) Data analysis ✓
	20c	Definition of analysis population relating to protocol non-adherence (eg, as randomised analysis), and any statistical methods to handle missing data (eg, multiple imputation) Data analysis ✓
**Methods: Monitoring**		
Data monitoring	21a	Composition of data monitoring committee (DMC); summary of its role and reporting structure; statement of whether it is independent from the sponsor and competing interests; and reference to where further details about its charter can be found, if not in the protocol. Alternatively, an explanation of why a DMC is not needed. Last paragraph of "Treatment allocation and safety monitoring" ✓
	21b	Description of any interim analyses and stopping guidelines, including who will have access to these interim results and make the final decision to terminate the trial Last paragraph of "Treatment allocation and safety monitoring" ✓
Harms	22	Plans for collecting, assessing, reporting, and managing solicited and spontaneously reported adverse events and other unintended effects of trial interventions or trial conduct- Treatment allocation and safety monitoring ✓
Auditing	23	Frequency and procedures for auditing trial conduct, if any, and whether the process will be independent from investigators and the sponsor "Role of the Sponsor"✓
**Ethics and dissemination**		
Research ethics approval	24	Plans for seeking research ethics committee/institutional review board (REC/IRB) approval – Ethics and dissemination ✓
Protocol amendments	25	Plans for communicating important protocol modifications (eg, changes to eligibility criteria, outcomes, analyses) to relevant parties (eg, investigators, REC/IRBs, trial participants, trial registries, journals, regulators) – "Trial monitoring and oversight" ✓
Consent or assent	26a	Who will obtain informed consent or assent from potential trial participants or authorised surrogates, and how (see Item 32) – Participant identification✓
	26b	Additional consent provisions for collection and use of participant data and biological specimens in ancillary studies, if applicable NA

only

Confidentiality	27	How personal information about potential and enrolled participants will be collected, shared, and maintained in order to protect confidentiality before, during, and after the trial – Section "data analysis"- ✓
Declaration of interests	28	Financial and other competing interests for principal investigators for the overall trial and each study site -Competing interests statement✓
Access to data	29	Statement of who will have access to the final trial dataset, and disclosure of contractual agreements that limit such access for investigators Section "data analysis"- ✓
Ancillary and post-trial care	30	Provisions, if any, for ancillary and post-trial care, and for compensation to those who suffer harm from trial participation – Last paragraph of "treatment allocation, blinding and safety monitoring" ✓
Dissemination policy	31a	Plans for investigators and sponsor to communicate trial results to participants, healthcare professionals, the public, and other relevant groups (eg, via publication, reporting in results databases, or other data sharing arrangements), including any publication restrictions Ethics and dissemination ✓
	31b	Authorship eligibility guidelines and any intended use of professional writers NA
	31c	Plans, if any, for granting public access to the full protocol, participant-level dataset, and statistical code NA
**Appendices**		
Informed consent materials	32	Model consent form and other related documentation given to participants and authorised surrogates
Biological specimens	33	Plans for collection, laboratory evaluation, and storage of biological specimens for genetic or molecular analysis in the current trial and for future use in ancillary studies, if applicable

*It is strongly recommended that this checklist be read in conjunction with the SPIRIT 2013 Explanation & Elaboration for important clarification on the items. Amendments to the protocol should be tracked and dated. The SPIRIT checklist is copyrighted by the SPIRIT Group under the Creative Commons "Attribution-NonCommercial-NoDerivs 3.0 Unported" license.

For peer review only

BMJ Open

Azathioprine immunosuppression and disease modification in Parkinson’s disease (AZA-PD): a randomised double-blind placebo-controlled phase II trial protocol

Journal:	*BMJ Open*
Manuscript ID	bmjopen-2020-040527.R1
Article Type:	Protocol
Date Submitted by the Author:	01-Sep-2020
Complete List of Authors:	Greenland, Julia; University of Cambridge, Department of Clinical Neurosciences<br>Cutting, Emma; University of Cambridge, Department of Clinical Neurosciences; Cambridge Clinical Trials Unit<br>Kadyan, Sonakshi; Cambridge Clinical Trials Unit<br>Bond, Simon; Cambridge Clinical Trials Unit<br>Chhabra, Anita; Cambridge University Hospitals NHS Foundation Trust, Pharmacy<br>Williams-Gray, Caroline; University of Cambridge, Department of Clinical Neurosciences
**Primary Subject Heading**:	Neurology
Secondary Subject Heading:	Pharmacology and therapeutics
Keywords:	Parkinson-s disease < NEUROLOGY, THERAPEUTICS, IMMUNOLOGY

SCHOLARONE™
Manuscripts

I, the Submitting Author has the right to grant and does grant on behalf of all authors of the Work (as defined in the below author licence), an exclusive licence and/or a non-exclusive licence for contributions from authors who are: i) UK Crown employees; ii) where BMJ has agreed a CC-BY licence shall apply, and/or iii) in accordance with the terms applicable for US Federal Government officers or employees acting as part of their official duties; on a worldwide, perpetual, irrevocable, royalty-free basis to BMJ Publishing Group Ltd ("BMJ") its licensees and where the relevant Journal is co-owned by BMJ to the co-owners of the Journal, to publish the Work in this journal and any other BMJ products and to exploit all rights, as set out in our licence.

The Submitting Author accepts and understands that any supply made under these terms is made by BMJ to the Submitting Author unless you are acting as an employee on behalf of your employer or a postgraduate student of an affiliated institution which is paying any applicable article publishing charge ("APC") for Open Access articles. Where the Submitting Author wishes to make the Work available on an Open Access basis (and intends to pay the relevant APC), the terms of reuse of such Open Access shall be governed by a Creative Commons licence – details of these licences and which Creative Commons licence will apply to this Work are set out in our licence referred to above.

Other than as permitted in any relevant BMJ Author's Self Archiving Policies, I confirm this Work has not been accepted for publication elsewhere, is not being considered for publication elsewhere and does not duplicate material already published. I confirm all authors consent to publication of this Work and authorise the granting of this licence.

For peer review only

Title

Azathioprine immunosuppression and disease modification in Parkinson's disease (AZA-PD): a randomised double-blind placebo-controlled phase II trial protocol

Authors

Julia C. Greenland[1], Emma Cutting[1,2], Sonakshi Kadyan[2], Simon Bond[2], Anita Chhabra[3], Caroline H. Williams-Gray[1]

[1.]Department of Clinical Neurosciences, John van Geest Centre for Brain Repair, University of Cambridge, Cambridge, UK.

[2]Cambridge Clinical Trials Unit, Cambridge University Hospitals NHS Foundation Trust, Cambridge

[3] Pharmacy, Cambridge University Hospitals NHS Foundation Trust, Cambridge

Corresponding author

Julia Greenland

John van Geest Centre for Brain Repair, Department of Clinical Neurosciences, University of Cambridge, Forvie Site, Cambridge CB2 0PY, United Kingdom.

Tel: +44 1223 331160

Fax: +44 1223 331174

Jcg69@cam.ac.uk

Keywords

Parkinson's disease, immune system, immunosuppression, clinical trial, therapy

Word Count

3951

For peer review only

For peer review only

Abstract

Introduction

The immune system is implicated in the aetiology and progression of Parkinson's disease (PD). Inflammation and immune activation occur both in the brain and in the periphery, and a pro-inflammatory cytokine profile is associated with more rapid clinical progression. Furthermore, the risk of developing PD is related to genetic variation in immune-related genes, and reduced by the use of immunosuppressant medication. We are therefore conducting a 'proof of concept' trial of azathioprine, an immunosuppressant medication, to investigate whether suppressing the peripheral immune system has a disease-modifying effect in PD.

Methods and analysis

AZA-PD is a phase II randomised placebo-controlled double-blind trial in early PD. Sixty participants, with clinical markers indicating an elevated risk of disease progression and no inflammatory or immune comorbidity, will be treated (azathioprine: placebo, 1:1) for 12 months, with a further 6-months follow-up. The primary outcome is the change in the MDS-UPDRS gait/axial score in the OFF state over the 12-month treatment period. Exploratory outcomes include additional measures of motor and cognitive function, non-motor symptoms and quality of life. In addition, peripheral and central immune markers will be investigated through analysis of blood, cerebrospinal fluid and PK-11195 PET imaging.

Ethics and dissemination

The study was approved by the London-Westminster research ethics committee (reference 19/LO/1705) and has been accepted by the MHRA for a clinical trials authorisation (reference CTA 12854/0248/001-0001). In addition, approval has been granted from the Administration of Radioactive Substances Advisory Committee (ARSAC). The results of this trial will be disseminated

For peer review only

through publication in scientific journals and presentation at national and international conferences and a lay summary will be available on our website.

Trial Registration

ISRCTN14616801, 14/5/2020. EudraCT- 2018-003089-14

Protocol version 1.1: AZA-PD CCTU0218

Strengths and limitations of this study

- First clinical trial of a peripherally acting immunosuppressive drug in Parkinson's disease
- Robust, randomised double-blind placebo-controlled design
- Novel patient stratification approach with recruitment of a more rapidly progressing subgroup to optimise chance of demonstrating clinical effect
- Detailed exploratory measures examining peripheral and central immune profile in PD to demonstrate proof of mechanism
- As a single centre 'proof of concept' trial, sample size is limited to 60 participants.

For peer review only

INTRODUCTION

Parkinson’s disease (PD) is a common neurodegenerative disorder diagnosed clinically by key motor features. The core pathology in PD involves the loss of dopaminergic neurons in the substantia nigra (SN) with intracellular accumulation of alpha-synuclein aggregates (Lewy bodies). Dopamine replacement therapy can control some of the motor symptoms. However, other problems including impaired balance and cognitive dysfunction are due to more widespread neurodegenerative pathology and are consequently unresponsive to dopaminergic therapies. These symptoms progress such that by 10 years from diagnosis, around two thirds of patients have balance and walking difficulties, and around half have dementia,[1] with a profound impact on quality of life [2, 3] and care requirements.[4] There are currently no treatments to alter disease course and prevent these devastating complications, hence there is an urgent need to find effective disease-modifying therapies for PD. There is increasing evidence that the immune system plays an important role in driving neurodegeneration in PD, and we propose that targeting the immune system may be an effective strategy for slowing disease progression.

The link between genetic variation in immune pathways and risk of Parkinson’s disease is well-established. Risk of developing PD is associated with polymorphisms in the Human Leucocyte Antigen (HLA) region, which encodes proteins vital to antigen recognition and presentation.[5, 6] Large scale analyses of Genome Wide Association Study (GWAS) data also implicate the immune system in PD, demonstrating heritability enrichment for genes of the adaptive immune system, including those involved in lymphocyte regulation and cytokine signalling pathways.[7, 8] Further evidence of an immune contribution to PD risk comes from epidemiological studies: individuals who regularly take non-steroidal anti-inflammatory drugs (NSAIDs) have reduced risk of developing PD [9, 10], as do those on immunosuppressive therapy.[11] There is also evidence that immune activation impacts on disease progression rate. In a large incident PD cohort, a pro-inflammatory serum cytokine profile at baseline

For peer review only

was associated with faster motor progression and impaired cognition over 36 months of follow-up.[12]

Activation of microglia, the inflammatory cells of the brain, has been clearly demonstrated in PD patients both at post-mortem [13-16] and using [^{11}C]-PK11195 positron emission tomography (PET) imaging in-vivo.[17-19] These cells have a role in responding to tissue injury, regulating the cerebral microenvironment, and antigen presentation.[20] It is thought that this activation is driven by toxic misfolded or post-translationally modified forms of alpha-synuclein released by degenerating cells, leading to secretion of proinflammatory and neurotoxic molecules, resulting in a cyclical process of cell damage.[21]

Abnormalities in the peripheral immune profile in PD are also well demonstrated and include alterations in both the innate and adaptive immune compartments. There is a shift towards 'classical' (inflammatory) monocytes with elevated expression of activation markers,[22] particularly in patients at higher dementia risk.[23] In the T lymphocyte compartment, several authors have reported bias towards pro-inflammatory CD4+ lymphocyte subsets and production of pro-inflammatory cytokines.[24-27] There may also be a reduction in the number and function of CD4+ T-regulatory (Treg) cells, whose role is to counter this pro-inflammatory response.[24, 26] In addition, changes in the CD8 compartment have been reported, with increased expression of activation markers and reduced markers of age-related senescence.[28]

Importantly, T-cells with specificity for epitopes of alpha-synuclein have been identified and reported to occur at higher frequency in PD than controls; furthermore their frequency was closely associated with possession of known PD risk alleles at the HLA locus[29], thus suggesting that alpha-synuclein may drive a peripheral adaptive immune response as well as an innate response of microglia in the brain. Elevated levels of alpha-synuclein specific antibodies are also present in the early stages of PD.[30] Peripheral immune cells may contribute to brain inflammation and neurotoxicity by trafficking into the central nervous system in PD. CD4+ and CD8+ lymphocytes have been shown to be present

For peer review only

in increased numbers in the SN at post-mortem in PD patients,[16, 25] as well as in ex-vivo cerebrospinal fluid (CSF) samples.[31] The precise mechanism by which peripheral immune cells drive neuronal damage in PD is still unclear, but it has recently been demonstrated that Th17 cells from PD patients drive cell death in autologous iPSC-derived dopaminergic midbrain neurons.[25]

Immune manipulation in animal models of PD alters disease susceptibility and severity. Using an MPTP mouse model of PD, studies have demonstrated that a lack of CD4+ lymphocytes attenuates dopaminergic cell death,[16] as does administration of Treg cells.[32] In mice that over-express alpha-synuclein, knockout of MHCII prevents both microglial activation and dopaminergic neurodegeneration.[33] Furthermore, cyclosporin, a widely used immunosuppressant, is effective in improving motor and cognitive deficits in multiple mouse models of PD.[34]

Although animal models of PD indicate that immunomodulatory therapies may have efficacy in protecting against neurodegeneration, there is limited clinical trial data in PD to date. Phase II trials of minocycline and pioglitazone, agents which reduce microglial activation in the brain in animal models, have been negative.[35-37] An early phase trial of sargramostim, a human recombinant granulocyte-macrophage colony-stimulating factor which promotes differentiation of pro-inflammatory T-effector cells into Treg cells has reported a modest improvement in an exploratory outcome of motor function over 8-weeks treatment.[38]

We propose that direct suppression of the peripheral immune system is an alternative, highly relevant therapeutic strategy which has not been tested in clinical trials to date. Azathioprine is an immunosuppressant drug widely used in clinical practice for a range of immune-related conditions. It is a purine analogue which inhibits nucleic acid synthesis, hence reducing proliferation of lymphocytes involved in targeting and amplification of the immune response. It affects both the cell-mediated and antibody-mediated responses through reducing T and B lymphocyte proliferation.[39] It was selected over other immunosuppressants because of its established efficacy in a range of clinical conditions, including central nervous system disorders such as multiple sclerosis,[40] and its acceptable safety

For peer review only

profile with recognised protocols for toxicity monitoring. Furthermore, it is generally well tolerated in the elderly and is a once-daily preparation for ease of administration.

METHODS AND ANALYSIS

Overview

AZA-PD is a randomised double-blind placebo-controlled trial of azathioprine in early Parkinson's disease which aims to provide 'proof of concept' that a peripherally acting immunosuppressive drug can slow clinical disease progression. The trial will investigate whether the drug has an effect on disease course over 12 months of treatment and whether this is maintained over 6 months of subsequent follow-up. Sixty participants will be recruited and randomised 1:1 to receive active treatment or placebo. Clinical assessments will be performed at baseline, 6 months, 12 months and 18 months (6 months post completion of treatment), with rigorous safety monitoring. In addition, the trial aims to demonstrate 'proof of mechanism' by evaluating the impact of azathioprine on blood, CSF and neuroimaging parameters of immune activation in the trial population and determining the relationship between these parameters and clinical measures of disease progression.

The trial timeline is summarised in **Error! Reference source not found.**.

Although AZA-PD is open to recruitment, given the current COVID-19 pandemic, recruitment has not commenced due to safety concerns. The Trial Steering Committee (TSC) and Data and Safety Monitoring Board (DSMB) will decide on an appropriate date to begin recruitment in due course, and protocol amendments to maximise patient safety will be submitted to the appropriate bodies when the best course of action has been determined. Our current aim is to start recruitment in March 2021, closing to recruitment in March 2022, with last patient last visit in November 2023, although this may be subject to change depending on the COVID-19 pandemic.

For peer review only

Patient and public involvement (PPI)

PD patients and their partners and carers who attend our research clinic at the John van Geest Centre for Brain Repair (VGB), University of Cambridge gave input into the protocol design. A PPI advisory panel of 4 patients/carers reviewed the protocol and provided specific feedback, leading to the addition of optional components. The PPI panel also reviewed our participant information sheet for clarity.

For peer review only

Participant Identification

Participants will be recruited from a single site in Cambridge, UK. Potential participants will be identified from the PD Research clinic database at the VGB. These individuals have undergone detailed clinical phenotyping, and information on demographics, comorbidities and medication is available. They have consented to be contacted about other research studies. Potential participants will be pre-selected by cross-referencing existing data with the inclusion/exclusion criteria outlined in **Error! Reference source not found.**. A key component of this process involves the calculation of a prognostic risk score, based on a model we have previously developed and validated, using age, MDS-UPDRS axial score and semantic fluency to estimate risk of a poor prognosis (dementia, postural instability or death) within 5 years.[41] Only patients with a risk greater than 50%, based on prior assessment at the research clinic, will be invited to take part. Approximately 40% of those on the database fall within this group. This strategy has been adopted to maximise the probability of demonstrating significant slowing of clinical progression with azathioprine treatment.

Potential participants will be sent a copy of the participant information sheet, and telephoned after two weeks to determine whether they are interested in participating. If so, they will attend a screening visit, where written informed consent will be obtained before confirming eligibility.

Eligibility criteria

A potential participant will be deemed eligible for recruitment into AZA-PD if they meet the inclusion and exclusion criteria listed in **Error! Reference source not found.**. A review of medical history and blood tests will be used to determine eligibility.

Outcome measures

The primary outcome measure is change in MDS-UPDRS gait/axial score in the OFF state over the 12-month treatment period. This is a clinical measure which has been shown to be the most sensitive measure of motor progression in PD, is relatively resistant to dopaminergic therapy and has an important impact on quality of life.[42] This score is a sum of the points from the following sections of MDS-UPDRS part III:

- 3.1 – speech
- 3.2 – facial expression
- 3.9 – rising from a chair
- 3.10 – gait
- 3.12 – postural stability
- 3.13 – posture
- 3.14 – body bradykinesia

Other outcome measures are exploratory and include:

- change in MDS-UPDRS gait/axial score in OFF state at 18 months
- change in total MDS-UPDRS in OFF state at 12 and 18 months
- change in electromagnetic sensor (EMS) measurements whilst performing MDS-UPDRS tremor and bradykinesia assessments at 12 and 18 months

For peer review only

- proportion of patients developing postural instability (Hoehn and Yahr stage 3 or greater) at 12 and 18 months
- change in global cognition (ACE-III) at 12 and 18 months
- change in patient reported outcome measure of quality of life (PDQ-39) at 12 and 18 months
- change in NMSS at 12 and 18 months
- change in dose of symptomatic dopaminergic therapy (LEDD) at 12 and 18 months
- the safety and tolerability of azathioprine assessed by the number of adverse events (AEs) recorded during the 12-month treatment period
- change in [^{11}C]-PK11195 PET non- dissociable binding potential (BP_{ND}) in subcortical and cortical regions of interest at 12 months
- change in total lymphocyte count at 6, 12 and 18 months
- change in serum immunoglobulin levels at 6, 12 and 18 months
- change in levels of serum and CSF cytokine levels at 12 and 18 months
- change in lymphocyte subsets in blood and CSF at 12 and 18 months

Sample Size Calculation

The treatment effect size is unknown and therefore cannot be used to inform sample size calculations. A sample size of 60 has been selected pragmatically based on feasibility of recruitment.

However, longitudinal clinical data from the ICICLE-PD cohort study provides some idea of an anticipated effect size for the primary outcome measure. ICICLE-PD patients were stratified by cytokine profile. Those with a 'pro-inflammatory' profile (n=32) had a more rapid symptom progression, with mean (SD) annualised change in MDS-UPDRS gait/axial score of 1.95 (1.92). In the subgroup with an 'anti-inflammatory' cytokine profile (n=26), mean (SD) annualised change in MDS-

For peer review only

UPDRS gait/axial score was 0.72 (1.40).[12] The corresponding between group difference of 1.2 points is equivalent to a standardised effect size (Cohen's d) of 0.73. The magnitude this effect, which equates to a 4% change on the 28-point gait-axial MDS-UPDRS subscale, would be clinically significant. For comparison, the estimated minimum clinically important change on the full 132-point MDS-UPDRS motor scale is ≈2% (2.5 points).[43] Furthermore, the axial-gait items of the MDS-UPDRS are those with the greatest impact on quality of life.[42]

As this is an early-phase proof of concept trial, it is important to maximise the chances of continuing development if the treatment is genuinely effective, and thus a significance level of 25% under a 1-sided test will be used. If the treatment effect is a 2% change (0.37 standardised effect), the design has 78% power, and for a 4% change (0.73 standardised effect), the design has 99% power.

Trial procedures

Clinical

Clinical measures assessing both motor and non-motor components of PD will be performed at baseline, mid-treatment, end-of-treatment and after 6-months further follow-up (see **Error! Reference source not found.**). Throughout the course of the trial participants will continue to take their PD medication as prescribed by their treating physician, and dose adjustments are permitted. However, some assessments will be conducted 'OFF' medication.

The Movement Disorder Society-Unified Parkinson's Disease Rating Scale (MDS-UPDRS) is widely used to quantify PD severity and includes questionnaires assessing the non-motor and motor aspects of the disease, a motor examination performed by a clinician and an assessment of motor complications (dyskinesias and fluctuations).[44] The MDS-UPDRS part III will be assessed in the OFF state; in the absence of regular dopaminergic medication, with participants being asked to not take their levodopa in the eight hours prior to the assessment or their long acting agents (e.g. ropinirole,

For peer review only

pramipexole, rasagiline) in the preceding 36 hours. The aim of this is to expose underlying disease severity and avoid confounding effects from variability in medication doses or timing. This examination will be filmed to enable subsequent rating by an independent assessor to check inter-rater reliability. Our primary outcome measure is derived from the MDS-UPDRS: the gait-axial sub-score, as previously discussed. Two sections of the MDS-UPDRS part III (tremor and bradykinesia) will be repeated whilst the participant is wearing an electromagnetic sensor (EMS, Polhemus Inc.) on the index finger and thumb, which will give an objective measure of the participant's movements. Motor stage will also be evaluated using the Hoehn and Yahr scale, a 5-point scale used to capture the stages of progression of PD, with stage 3 representing the development of postural instability.[45]

Cognition will be assessed using the Addenbrooke's Cognitive Examination-III (ACE-III). This provides a global measure of cognition as well as sub-scores in 5 domains; attention, memory, fluency, language and visuospatial function.[46] Other non-motor aspects of PD will be evaluated using the short form 15-item Geriatric Depression Scale (GDS), a questionnaire assessing depressive symptoms filled in by the participant,[47] and the Parkinson's disease Non-Motor Symptom Scale (NMSS), completed by the trial assessor.[48] Finally, we will use the Parkinson's Disease Questionnaire 39 (PDQ-39), a self-rated questionnaire measuring PD-related quality of life.[49]

Dopaminergic medication requirement will be monitored throughout the trial, and standardised by calculating Levodopa Equivalent Daily Dose (LEDD), which allows quantification of different doses and types of Parkinson's medication on a single scale.[50]

PK-11195 PET imaging

$[^{11}C]$-PK11195 PET will be used to measure activated microglia in the brain.[17, 18] Scanning will be conducted at the Wolfson Brain Imaging Centre (WBIC) on a GE SIGNA PET/MRI scanner, with the

For peer review only

radiotracer produced at the WBIC Radiopharmaceutical Chemistry laboratory. MRI will be used for co-localisation. 500 MBq of the[^{11}C]-PK11195 radiotracer will be injected via a peripheral venous cannula over 30 seconds and PET emission data will be acquired for 75 min post-injection in 55 time frames. Following image reconstruction and attenuation correction, specific tracer binding will be analysed with the simplified reference tissue model (SRTM; [51]) to quantify binding potential relative to a non-displaceable compartment (BPND). The reference region will be estimated with supervised cluster analysis for [^{11}C]PK11195 from existing scans in healthy controls acquired on the same scanner. BPND will be compared pre and post treatment using a region of interest approach.

Given that some participants may have difficulty tolerating prolonged PET imaging, this will be optional. It will be performed between screening and baseline, and repeated within 3 months following the end of treatment.

Biosample collection and processing

14 mls of blood will be collected in serum tubes at baseline, mid-treatment, end-of-treatment and follow-up visits for analysis of inflammatory cytokines, CRP and immunoglobulins. Tubes will be centrifuged at 2000RPM (600G) for 15 mins following 15 minutes clotting time for extraction of serum. Aliquots will be stored at -80°C for subsequent batch analysis using ELISA and electrochemiluminescence assays.

At baseline, end-of-treatment and follow-up visits an additional 27mls of blood will be collected in lithium heparin tubes for separation of peripheral blood mononuclear cells (PBMCs) for immunophenotyping. A concurrent full blood count (FBC) will be performed from an EDTA sample (2.6mls).

CSF will be collected via lumbar puncture before the baseline visit and at the treatment endpoint. This is an optional component of the study in order to ensure that its inclusion does not limit recruitment. CSF will be spun at 400G for 10 minutes for extraction of immune cells for

For peer review only

contemporaneous immunophenotyping alongside PBMC analysis. Supernatants will be stored at -80°C for later batch analysis of relevant immune and protein markers. Immunophenotyping will be performed for subsets of T cells, B cells and monocytes using flow cytometry, run within 24 hours of sample collection.

For peer review only

Table 1: Schedule of Assessments

		Screening Visit<br>Day -42 (max)	Imaging Visit<br>Approx. Day -14±	CSF Collection<br>Approx. Day -7±	Baseline Visit<br>Day 0± 14	Monitoring Visits[1]<br>Day 14 ± 5 and onwards	Dose escalation Visit<br>Day 28± 5	Mid-Treatment Visit<br>Day 182± 14	End of Treatment Visit<br>Day 365± 14	Imaging Visit<br>Day 410± 45	Follow-up Visit<br>Day 547±14
	Informed written consent	✓									
	Eligibility review	✓									
	Randomisation				✓						
	Vital Signs				✓	✓	✓	✓	✓		✓
	Weight in kg	✓			✓	✓	✓	✓			
Clinical Assessments	Demographics				✓						
	Medical history	✓			✓	✓	✓	✓	✓		✓
	Concomitant medication review	✓			✓	✓	✓	✓	✓		✓
	MDS-UPDRS				✓			✓	✓		✓
	NART				✓						
	ACE III				✓			✓	✓		✓
	GDS				✓			✓	✓		✓
	NMSS				✓			✓	✓		✓
	PDQ-39				✓			✓	✓		✓
	Adverse events review	✓	✓	✓	✓	✓	✓	✓	✓	✓	✓
	IMP compliance check					✓	✓	✓	✓		

Imaging-Optional	[11C]-PK11195 PET-MRI		✓							✓	
Blood tests	Screening bloods[2]	✓									
	Safety monitoring bloods[3]					✓	✓	✓	✓		✓
	CRP, immunoglobulins and serum storage for cytokine measurement			✓				✓	✓		✓
	Immunophenotyping			✓					✓		✓
CSF-optional	Immune markers and immunophenotyping			✓					✓		

[1] Monitoring visits will take place at: day 14, day 42, day 56, day 70, day 98, day 252 and day 547 (as part of the routine follow-up visit). Additional monitoring visits may also be scheduled if there are patient safety concerns.

[2]Screening bloods include FBC, U&Es, LFTs, coagulation, TPMT, HIV, Syphilis, HepB, HepC, EBV serology, VZV serology, LH and FSH (if female and reproductive age)

[3]Monitoring bloods include FBC, U&Es, LFTs

For peer review only

Treatment allocation, blinding and safety monitoring

Participants will be randomised 1:1 to receive azathioprine or placebo using Sealed Envelope, an online randomisation system. Clinical assessors and participants will be blinded to treatment allocation. Balanced assignment of each treatment will be achieved using permuted block randomisation, which will be stratified for: age ≤ 71 vs > 71, and MDS-UPDRS-III ≤ 30 vs > 30.

Treatment will be commenced at a dose of 1mg/kg, based on 25mg tablets of IMP (azathioprine/placebo). In addition to the visits shown in *Figure 1*, treatment monitoring visits will be conducted to screen for potential complications associated with azathioprine. These will include blood tests to screen for myelosuppression, liver or renal dysfunction, adverse events reporting and assessment of treatment compliance (review of patient-completed dosing diary and counting of IMP at regular intervals). Initially, monitoring visits will occur 2 weekly, and after 4 weeks, the azathioprine dose will be increased to 2mg/kg (assuming blood tests and clinical assessments are satisfactory), the standard therapeutic dose used in clinical practice. There will be a matched doubling of the placebo dose to maintain blinding. Once the participant is stable on their dose, treatment monitoring will be carried out less frequently (see monitoring protocol, *Figure 3*).

Given that azathioprine will produce changes in FBC parameters, the blinded trial team conducting patient assessments and laboratory analysis will not have access to monitoring blood results throughout the duration of the trial. The blood tests will be reviewed by a separate unblinded team of clinicians, who will make decisions on dose changes when necessary. If a dose reduction is required, the participant will have an additional 3 monitoring visits at 2-weekly intervals to ensure stability of blood tests. Dose reductions and, where necessary, withdrawal of treatment will be carried out based on pre-defined clinical and laboratory criteria to ensure the safety of participants, including the development of significant myelosuppression, intolerable gastrointestinal side effects and hypersensitivity reactions. Participants who have been withdrawn from treatment will be encouraged to continue to attend the remainder of the trial assessments.

For peer review only

To ensure blinding is maintained amongst clinical assessors and participants, dose adjustments and treatment withdrawals will be also made for an equal number of participants in the placebo arm, with additional monitoring visits. Matched pairs of placebo and azathioprine-treated participants will be generated to facilitate this, and all dose adjustment decisions will be made by the unblinded team.

Emergency unblinding will be carried out in the event of a valid medical or safety reason, where the clinical care of the participant will be facilitated by the knowledge of whether they have been taking azathioprine, as decided by the treating clinician. It will be executed using Sealed Envelope, and where possible the trial team will remain blinded.

Following the end of the trial, and for participants who withdraw early, we will offer continuing follow-up through our research clinic at the VGB.

Trial monitoring and oversight

Safety monitoring will be overseen by a DSMB who will have access to interim recruitment and safety data. The DSMB will report to the TSC should it become clear that one treatment allocation is either indicated or contraindicated, or apparent that no clear outcome can be obtained from the trial. The TSC, who are independent from the Sponsor, will provide overall supervision of the trial and ensure that it meets appropriate standards. These groups include clinicians with experience in PD or immunosuppression, independent statisticians, and the TSC includes a lay member.

AZA-PD is jointly sponsored by Cambridge University Hospitals NHS Foundation Trust and the University of Cambridge. The Sponsor will review all trial documentation, including any proposed amendments, prior to submission to the relevant regulatory bodies, which can only be completed

For peer review only

once the Sponsor has approved the changes. Changes will then be communicated to participants, the DMSB, TSC, and trial registries.

Adherence to the protocol and regulatory requirements will be reviewed by a Clinical Trials Monitor, assigned by the Sponsor. The first monitoring visit will occur within 10 days of the first randomisation, with frequency thereafter determined by a risk assessment which will be reviewed and adjusted as necessary throughout the course of the trial.

Data Analysis

Trial data will be transferred from paper CRFs to the electronic trial database, where it will be anonymised, but with preserved linkage records. Patient-identifiable data (PID), will be stored on a password-protected database within the SDHS hosted by the University of Cambridge, with access granted only to relevant members of the trial team. PID will be kept for 5 years following the end of the trial, as per regulatory requirements. Participant consent will be specifically sought for data/sample sharing with our collaborators, and use of remaining biological samples in future ethically-approved research.

Data will be analysed on an "intention to treat" basis, with further "per protocol" analysis in participants with at least 80% compliance with trial medication. All endpoints will be summarised and broken down by treatment group and time point, where relevant. Mean, median, standard deviation, minimum/maximum will be used for continuous endpoints, and frequency tables for categorical or binary endpoints. Equivalent box and whisker plots or stacked bar charts will be produced for continuous and categorical endpoints respectively.

The primary analysis will estimate the difference between treatment groups in terms of the primary endpoint. An analysis of covariance (ANCOVA) model will be fitted adjusting for baseline MDS-UPDRS gait/axial score, gender, LEDD, and age. Treatment effect estimates, standard errors, confidence

For peer review only

intervals (95% and 40% levels) and 1-sided p-values will be provided. A 1-sided p-value less than 25% will be regarded as statistically significant. Similar comparative analyses will be produced for other time points of the MDS-UPDRS gait/axial score and exploratory endpoints, using ANCOVA for continuous endpoints or logistic regression for categorical or binary endpoints.

Longitudinal data will use a mixed effect model repeated measurements (MMRM) analysis, to include an unstructured patient-level random effect for nominal visit, visit and visit-treatment interaction fixed effects at visits post-baseline, with adjustment for baseline covariates. To assess the slope of change over time, the longitudinal data will be analysed using a similar MMRM but with a fixed effect of time from randomisation as a continuous, rather than nominal covariate, with a treatment-time interaction to compare treatment groups and patient-level random effect for slope, with adjustment for baseline covariates.

ETHICS AND DISSEMINATION

This study was approved by the London-Westminster research ethics committee (reference 19/LO/1705) and has been accepted by the MHRA for a clinical trials authorisation (reference CTA 12854/0248/001-0001). In addition, approval has been granted from the Administration of radioactive substances advisory committee (ARSAC).

We will feedback trial results to participants and our wider cohort of research participants via our annual PD Open Day and newsletter. A lay summary of the results will be available on our website. The results will also be disseminated through publication in scientific journals and presentation at national and international conferences.

AZA-PD has been accepted onto the NIHR Clinical Research Network (CRN) portfolio and details of this trial are also available on the following registries: ISRCTN14616801, EudraCT- 2018-003089-14.

For peer review only

REFERENCES

1. Williams-Gray CH, Mason SL, Evans JR, et al. The CamPaIGN study of Parkinson's disease: 10-year outlook in an incident population-based cohort. *Journal of neurology, neurosurgery, and psychiatry* 2013;84(11):1258-64. doi: 10.1136/jnnp-2013-305277 [published Online First: 2013/06/20]
2. Schrag A, Jahanshahi M, Quinn N. What contributes to quality of life in patients with Parkinson's disease? *Journal of neurology, neurosurgery, and psychiatry* 2000;69(3):308-12. [published Online First: 2000/08/17]
3. Evans JR, Mason SL, Williams-Gray CH, et al. The natural history of treated Parkinson's disease in an incident, community based cohort. *Journal of neurology, neurosurgery, and psychiatry* 2011;82(10):1112-8. doi: 10.1136/jnnp.2011.240366 [published Online First: 2011/05/20]
4. Aarsland D, Larsen JP, Tandberg E, et al. Predictors of nursing home placement in Parkinson's disease: a population-based, prospective study. *Journal of the American Geriatrics Society* 2000;48(8):938-42. [published Online First: 2000/09/01]
5. Saiki M, Baker A, Williams-Gray CH, et al. Association of the human leucocyte antigen region with susceptibility to Parkinson's disease. *Journal of neurology, neurosurgery, and psychiatry* 2010;81(8):890-1. doi: 10.1136/jnnp.2008.162883 [published Online First: 2010/05/14]
6. International Parkinson Disease Genomics Consortium, Nalls MA, Plagnol V, et al. Imputation of sequence variants for identification of genetic risks for Parkinson's disease: a meta-analysis of genome-wide association studies. *Lancet* 2011;377(9766):641-9.
7. Gagliano SA, Pouget JG, Hardy J, et al. Genomics implicates adaptive and innate immunity in Alzheimer's and Parkinson's diseases. *Annals of clinical and translational neurology* 2016;3(12):924-33. doi: 10.1002/acn3.369 [published Online First: 2017/01/18]

For peer review only

8. Holmans P, Moskvina V, Jones L, et al. A pathway-based analysis provides additional support for an immune-related genetic susceptibility to Parkinson's disease. *Human molecular genetics* 2013;22(5):1039-49. doi: 10.1093/hmg/dds492 [published Online First: 2012/12/12]

9. Gao X, Chen H, Schwarzschild MA, et al. Use of ibuprofen and risk of Parkinson disease. *Neurology* 2011;76(10):863-9. doi: 10.1212/WNL.0b013e31820f2d79 [published Online First: 2011/03/04]

10. Chen H, Zhang SM, Hernan MA, et al. Nonsteroidal anti-inflammatory drugs and the risk of Parkinson disease. *Archives of neurology* 2003;60(8):1059-64. doi: 10.1001/archneur.60.8.1059 [published Online First: 2003/08/20]

11. Racette BA, Gross A, Vouri SM, et al. Immunosuppressants and risk of Parkinson disease. *Annals of clinical and translational neurology* 2018;5(7):870-75. doi: 10.1002/acn3.580 [published Online First: 2018/07/17]

12. Williams-Gray CH, Wijeyekoon R, Yarnall AJ, et al. Serum immune markers and disease progression in an incident Parkinson's disease cohort (ICICLE-PD). *Movement disorders : official journal of the Movement Disorder Society* 2016;31(7):995-1003. doi: 10.1002/mds.26563

13. McGeer PL, Itagaki S, Boyes BE, et al. Reactive microglia are positive for HLA-DR in the substantia nigra of Parkinson's and Alzheimer's disease brains. *Neurology* 1988;38(8):1285-91. [published Online First: 1988/08/01]

14. Mogi M, Harada M, Kondo T, et al. Interleukin-1 beta, interleukin-6, epidermal growth factor and transforming growth factor-alpha are elevated in the brain from parkinsonian patients. *Neuroscience letters* 1994;180(2):147-50. [published Online First: 1994/10/24]

15. Loeffler DA, Camp DM, Conant SB. Complement activation in the Parkinson's disease substantia nigra: an immunocytochemical study.

For peer review only

Journal of neuroinflammation 2006;3:29. doi: 10.1186/1742-2094-3-29 [published Online First: 2006/10/21]

16. Brochard V, Combadiere B, Prigent A, et al. Infiltration of CD4+ lymphocytes into the brain contributes to neurodegeneration in a mouse model of Parkinson disease. *The Journal of clinical investigation* 2009;119(1):182-92. doi: 10.1172/jci36470 [published Online First: 2008/12/24]
17. Gerhard A, Pavese N, Hotton G, et al. In vivo imaging of microglial activation with [11C](R)-PK11195 PET in idiopathic Parkinson's disease. *Neurobiology of disease* 2006;21(2):404-12. doi: 10.1016/j.nbd.2005.08.002 [published Online First: 2005/09/27]
18. Ouchi Y, Yoshikawa E, Sekine Y, et al. Microglial activation and dopamine terminal loss in early Parkinson's disease. *Annals of neurology* 2005;57(2):168-75. doi: 10.1002/ana.20338 [published Online First: 2005/01/26]
19. Edison P, Ahmed I, Fan Z, et al. Microglia, amyloid, and glucose metabolism in Parkinson's disease with and without dementia. *Neuropsychopharmacology : official publication of the American College of Neuropsychopharmacology* 2013;38(6):938-49. doi: 10.1038/npp.2012.255 [published Online First: 2013/01/11]
20. Perry VH. Innate inflammation in Parkinson's disease. *Cold Spring Harbor perspectives in medicine* 2012;2(9):a009373. doi: 10.1101/cshperspect.a009373 [published Online First: 2012/09/07]
21. Gao HM, Hong JS. Why neurodegenerative diseases are progressive: uncontrolled inflammation drives disease progression. *Trends in immunology* 2008;29(8):357-65. doi: 10.1016/j.it.2008.05.002 [published Online First: 2008/07/05]
22. Grozdanov V, Bliederhaeuser C, Ruf WP, et al. Inflammatory dysregulation of blood monocytes in Parkinson's disease patients. *Acta neuropathologica* 2014;128(5):651-63. doi: 10.1007/s00401-014-1345-4

For peer review only

23. Wijeyekoon RS, Kronenberg-Versteeg D, Scott KM, et al. Peripheral innate immune and bacterial signals relate to clinical heterogeneity in Parkinson's disease. *Brain, behavior, and immunity* 2020 doi: 10.1016/j.bbi.2020.01.018 [published Online First: 2020/02/02]

24. Kustrimovic N, Comi C, Magistrelli L, et al. Parkinson's disease patients have a complex phenotypic and functional Th1 bias: cross-sectional studies of CD4+ Th1/Th2/T17 and Treg in drug-naive and drug-treated patients. *Journal of neuroinflammation* 2018;15(1):205. doi: 10.1186/s12974-018-1248-8 [published Online First: 2018/07/14]

25. Sommer A, Maxreiter F, Krach F, et al. Th17 Lymphocytes Induce Neuronal Cell Death in a Human iPSC-Based Model of Parkinson's Disease. *Cell stem cell* 2018;23(1):123-31.e6. doi: 10.1016/j.stem.2018.06.015 [published Online First: 2018/07/07]

26. Saunders JA, Estes KA, Kosloski LM, et al. CD4+ regulatory and effector/memory T cell subsets profile motor dysfunction in Parkinson's disease. *Journal of neuroimmune pharmacology : the official journal of the Society on NeuroImmune Pharmacology* 2012;7(4):927-38. doi: 10.1007/s11481-012-9402-z

27. Baba Y, Kuroiwa A, Uitti RJ, et al. Alterations of T-lymphocyte populations in Parkinson disease. *Parkinsonism & related disorders* 2005;11(8):493-8. doi: 10.1016/j.parkreldis.2005.07.005 [published Online First: 2005/09/13]

28. Williams-Gray CH, Wijeyekoon RS, Scott KM, et al. Abnormalities of age-related T cell senescence in Parkinson's disease. *Journal of Neuroinflammation* 2018;15:166. doi: 10.1186/s12974-018-1206-5

29. Sulzer D, Alcalay RN, Garretti F, et al. T cells from patients with Parkinson's disease recognize alpha-synuclein peptides. *Nature* 2017;546(7660):656-61. doi: 10.1038/nature22815 [published Online First: 2017/06/22]

For peer review only

30. Scott KM, Kouli A, Yeoh SL, et al. A Systematic Review and Meta-Analysis of Alpha Synuclein Auto-Antibodies in Parkinson's Disease. *Frontiers in neurology* 2018;9:815. doi: 10.3389/fneur.2018.00815 [published Online First: 2018/10/20]
31. Schroder JB, Pawlowski M, Meyer Zu Horste G, et al. Immune Cell Activation in the Cerebrospinal Fluid of Patients With Parkinson's Disease. *Frontiers in neurology* 2018;9:1081. doi: 10.3389/fneur.2018.01081 [published Online First: 2019/01/09]
32. Reynolds AD, Stone DK, Hutter JA, et al. Regulatory T cells attenuate Th17 cell-mediated nigrostriatal dopaminergic neurodegeneration in a model of Parkinson's disease. *Journal of immunology (Baltimore, Md : 1950)* 2010;184(5):2261-71. doi: 10.4049/jimmunol.0901852 [published Online First: 2010/02/02]
33. Harms AS, Cao S, Rowse AL, et al. MHCII is required for alpha-synuclein-induced activation of microglia, CD4 T cell proliferation, and dopaminergic neurodegeneration. *The Journal of neuroscience : the official journal of the Society for Neuroscience* 2013;33(23):9592-600. doi: 10.1523/jneurosci.5610-12.2013 [published Online First: 2013/06/07]
34. Tamburrino A, Churchill MJ, Wan OW, et al. Cyclosporin promotes neurorestoration and cell replacement therapy in pre-clinical models of Parkinson's disease. *Acta neuropathologica communications* 2015;3:84. doi: 10.1186/s40478-015-0263-6 [published Online First: 2015/12/17]
35. Investigators NN-P. A pilot clinical trial of creatine and minocycline in early Parkinson disease: 18-month results. *Clin Neuropharmacol* 2008;31(3):141-50. doi: https://dx.doi.org/10.1097/WNF.0b013e3181342f32
36. NINDS Net-Pd Investigators. A randomized, double-blind, futility clinical trial of creatine and minocycline in early Parkinson disease. *Neurology* 2006;66(5):664-71. doi: 10.1212/01.wnl.0000201252.57661.e1

37. Investigators NETiPDF-Z. Pioglitazone in early Parkinson's disease: a phase 2, multicentre, double-blind, randomised trial.[Erratum appears in Lancet Neurol. 2015 Oct; 14(10):979]. *Lancet neurol* 2015;14(8):795-803. doi: https://dx.doi.org/10.1016/S1474-4422(15)00144-1

38. Gendelman HE, Zhang Y, Santamaria P, et al. Evaluation of the safety and immunomodulatory effects of sargramostim in a randomized, double-blind phase 1 clinical Parkinson's disease trial. *NPJ Parkinson's disease* 2017;3:10. doi: 10.1038/s41531-017-0013-5 [published Online First: 2017/06/27]

39. Lennard L. The clinical pharmacology of 6-mercaptopurine. *European journal of clinical pharmacology* 1992;43(4):329-39. doi: 10.1007/bf02220605 [published Online First: 1992/01/01]

40. Casetta I, Iuliano G, Filippini G. Azathioprine for multiple sclerosis. *The Cochrane database of systematic reviews* 2007(4):Cd003982. doi: 10.1002/14651858.CD003982.pub2 [published Online First: 2007/10/19]

41. Velseboer DC, de Bie RM, Wieske L, et al. Development and external validation of a prognostic model in newly diagnosed Parkinson disease. *Neurology* 2016;86(11):986-93. doi: 10.1212/wnl.0000000000002437 [published Online First: 2016/02/19]

42. Evans JR, Mason SL, Williams-Gray CH, et al. The factor structure of the UPDRS as an index of disease progression in Parkinson's disease. *Journal of Parkinson's disease* 2011;1(1):75-82. doi: 10.3233/jpd-2011-0002 [published Online First: 2011/01/01]

43. Shulman LM, Gruber-Baldini AL, Anderson KE, et al. The clinically important difference on the unified Parkinson's disease rating scale. *Archives of neurology* 2010;67(1):64-70. doi: 10.1001/archneurol.2009.295

44. Goetz CG, Tilley BC, Shaftman SR, et al. Movement Disorder Society-sponsored revision of the Unified Parkinson's Disease Rating Scale

For peer review only

(MDS-UPDRS): scale presentation and clinimetric testing results. *Movement disorders : official journal of the Movement Disorder Society* 2008;23(15):2129-70. doi: 10.1002/mds.22340

45. Hoehn MM, Yahr MD. Parkinsonism: onset, progression and mortality. *Neurology* 1967;17(5):427-42. [published Online First: 1967/05/01]
46. Hsieh S, Schubert S, Hoon C, et al. Validation of the Addenbrooke's Cognitive Examination III in frontotemporal dementia and Alzheimer's disease. *Dementia and geriatric cognitive disorders* 2013;36(3-4):242-50. doi: 10.1159/000351671 [published Online First: 2013/08/21]
47. Sheikh JI YJ. Geriatric Depression Scale (GDS): recent evidence and development of a shorter version. *Clin Gerontol* 1986;5(June):165-73.
48. Chaudhuri KR, Martinez-Martin P, Brown RG, et al. The metric properties of a novel non-motor symptoms scale for Parkinson's disease: Results from an international pilot study. *Movement disorders : official journal of the Movement Disorder Society* 2007;22(13):1901-11. doi: 10.1002/mds.21596
49. Jenkinson C, Fitzpatrick R, Peto V, et al. The Parkinson's Disease Questionnaire (PDQ-39): development and validation of a Parkinson's disease summary index score. *Age and ageing* 1997;26(5):353-7. [published Online First: 1997/11/14]
50. Tomlinson CL, Stowe R, Patel S, et al. Systematic review of levodopa dose equivalency reporting in Parkinson's disease. *Movement disorders : official journal of the Movement Disorder Society* 2010;25(15):2649-53. doi: 10.1002/mds.23429 [published Online First: 2010/11/12]
51. Lammertsma AA, Hume SP. Simplified reference tissue model for PET receptor studies. *NeuroImage* 1996;4(3 Pt 1):153-8. doi: 10.1006/nimg.1996.0066 [published Online First: 1996/12/01]

For peer review only

Acknowledgements

This research was supported by the National Institute for Health Research (NIHR) Cambridge Biomedical Research Centre and the Cambridge Clinical Trials Unit (CCTU).

Authors' Contributions

CWG is the CI of this trial. JG is sub-investigator of this trial.

CWG and JG – study design and writing the protocol.

SK, EC and AC – critical review of the protocol.

SB - statistical analysis plan.

Sponsorship

This study is jointly sponsored by the University of Cambridge and Cambridge University Hospitals NHS foundation Trust.

Contact:

Ms Carrie Bayliss

Cambridge Clinical Trials Unit

Cambridge University Hospitals NHSFT

Hills Road

Cambridge

CB2 0QQ

E-mail: cctu@addenbrookes.nhs.uk

For peer review only

Funding Statement

This trial is funded by Cambridge Centre for Parkinson-Plus (grant no: RG95450) and the Cure Parkinson's Trust (grant ref CW011), and supported by the NIHR Cambridge Biomedical Research Centre (grant ref no 146281). CHWG is supported by a RCUK/UKRI Research Innovation Fellowship awarded by the Medical Research Council (MR/R007446/1).

The funders have no role in study design; collection, management, analysis, and interpretation of data; writing of the report; or the decision to submit the report for publication.

Competing Interests Statement

None declared

For peer review only

TABLE OF FIGURES

Figure 1: Overview of trial timeline

Figure 2: AZA-PD eligibility criteria

Figure 3: Treatment monitoring schedule

For peer review only

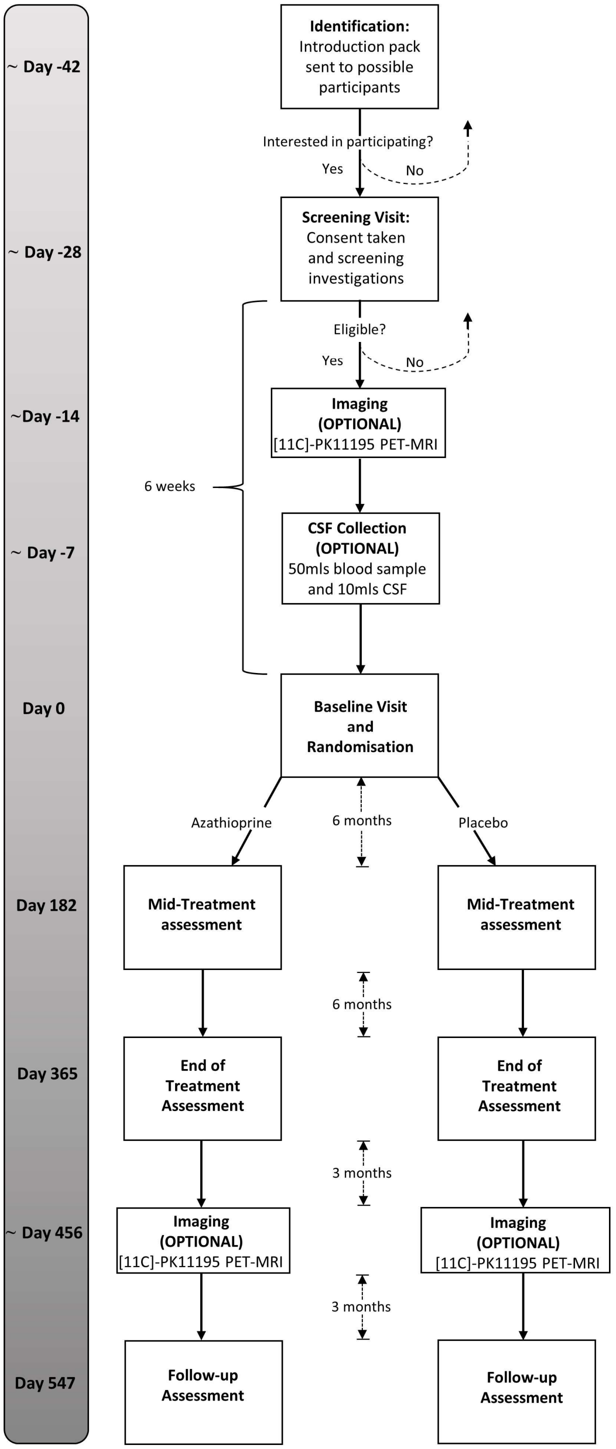
~ Day -42
~ Day -28
~Day -14
~ Day -7
Day 0
Day 182
Day 365
~ Day 456
Day 547
Identification: Introduction pack sent to possible participants
Interested in participating?
Yes
No
Screening Visit: Consent taken and screening investigations
Eligible?
Yes
No
Imaging (OPTIONAL) [11C]-PK11195 PET-MRI
6 weeks
CSF Collection (OPTIONAL) 50mls blood sample and 10mls CSF
Baseline Visit and Randomisation
Azathioprine
6 months
Placebo
Mid-Treatment assessment
Mid-Treatment assessment
6 months
End of Treatment Assessment
End of Treatment Assessment
3 months
Imaging (OPTIONAL) [11C]-PK11195 PET-MRI
Imaging (OPTIONAL) [11C]-PK11195 PET-MRI
3 months
Follow-up Assessment
Follow-up Assessment

Inclusion Criteria

- be capable of giving signed informed consent
- be aged over 50 years
- be a fluent English speaker
- have a diagnosis of PD according to UKPDS Brain Bank Criteria
- have a disease duration of less than 3 years
- have a probability of poor outcome (postural instability/dementia/death) at 5 years from diagnosis ≥50% [41]
- have adequate organ and marrow function, as defined below (measured within 42 days of first dose of trial medication):
    - Haemoglobin ≥ 110 g/L
    - Platelet count ≥ 130 x 10^9/L
    - Neutrophil count ≥ 1.5 x 10^9/L
    - Renal function- creatinine clearance ≥50mL/min.
    - Hepatic function- ALT and bilirubin ≤2 times the institutional upper limit of normal

Exclusion Criteria

- Any use of immunomodulatory drugs such as azathioprine, mycophenolate, methotrexate, ciclosporin, cyclophosphamide within the 12 months prior to screening
- Any previous use of rituximab or alemtuzumab at any time
- Treatment with oral corticosteroids for greater than 2 weeks within the 12 months prior to screening, or any oral steroid use in 3 months prior to screening
- Regular use of NSAIDs including aspirin >75mg, naproxen, ibuprofen, meloxicam on more than 2 days per week
- Known inflammatory or autoimmune disease
- Chronic or latent infection
- Active infection requiring the use of parenteral antimicrobial agents within 2 months prior to the first dose of trial treatment
- Skin or solid organ malignancy within the 5 years prior to the screening assessment
- Current or previous haematological malignancy
- The inability to take or swallow oral medication
- Parkinson's Disease Dementia according to MDS PD Dementia criteria
- A positive test for HIV or Hepatitis
- TPMT deficiency
- A lack of immunity to VZV
- Negative EBV IgG
- Chronic liver disease
- Renal impairment - creatinine clearance <50mL/min
- Current or previous haematological malignancy
- Concomitant allopurinol
- Any concurrent medical or psychiatric condition or disease that is likely to interfere with the trial procedures or results, or that in the opinion of the investigator, would constitute a hazard for participating in this trial
- Receipt of live, attenuated vaccine within the 30 days prior to the screening assessment
- Women of childbearing potential. Female patients must be surgically sterile or be postmenopausal
- Male patients must be surgically sterile or must agree to use effective contraception during the period of therapy and for 6 months after the last dose of the trial treatment
- Known hypersensitivity to azathioprine or its excipient
- Received an investigational drug or used an invasive investigational medical device within 4 weeks before the screening assessment or is currently enrolled in an interventional investigational trial

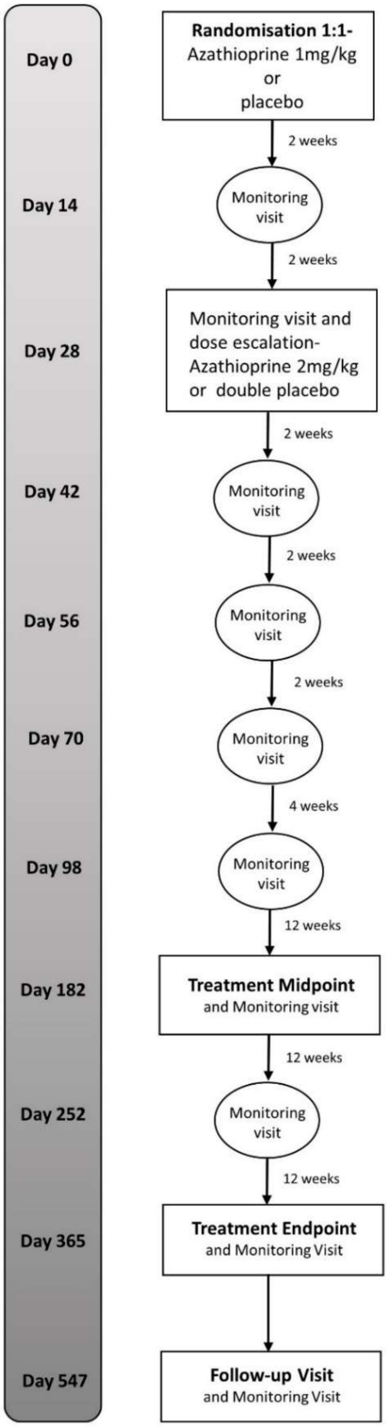
Day 0
Day 14
Day 28
Day 42
Day 56
Day 70
Day 98
Day 182
Day 252
Day 365
Day 547
Randomisation 1:1-
Azathioprine 1mg/kg
or
placebo
2 weeks
Monitoring visit
2 weeks
Monitoring visit and dose escalation- Azathioprine 2mg/kg or double placebo
2 weeks
Monitoring visit
2 weeks
Monitoring visit
2 weeks
Monitoring visit
4 weeks
Monitoring visit
12 weeks
Treatment Midpoint
and Monitoring visit
12 weeks
Monitoring visit
12 weeks
Treatment Endpoint
and Monitoring Visit
Follow-up Visit
and Monitoring Visit

SPIRIT

STANDARD PROTOCOL ITEMS: RECOMMENDATIONS FOR INTERVENTIONAL TRIALS

SPIRIT 2013 Checklist: Recommended items to address in a clinical trial protocol and related documents*

Section/item	Item No	Description
**Administrative information**		
Title	1	Descriptive title identifying the study design, population, interventions, and, if applicable, trial acronym ✓
Trial registration	2a	Trial identifier and registry name. If not yet registered, name of intended registry ✓
	2b	All items from the World Health Organization Trial Registration Data Set ✓
Protocol version	3	Date and version identifier ✓
Funding	4	Sources and types of financial, material, and other support "funding statement" ✓
Roles and responsibilities	5a	Names, affiliations, and roles of protocol contributors "Authors' contribution" ✓
	5b	Name and contact information for the trial sponsor "Sponsor contact"✓
	5c	Role of study sponsor and funders, if any, in study design; collection, management, analysis, and interpretation of data; writing of the report; and the decision to submit the report for publication, including whether they will have ultimate authority over any of these activities "Trial monitoring and oversight" and "Funding statement"✓
	5d	Composition, roles, and responsibilities of the coordinating centre, steering committee, endpoint adjudication committee, data management team, and other individuals or groups overseeing the trial, if applicable (see Item 21a for data monitoring committee) – safety monitoring and authors' contributions✓
**Introduction**		
Background and rationale	6a	Description of research question and justification for undertaking the trial, including summary of relevant studies (published and unpublished) examining benefits and harms for each intervention – covered in the introduction ✓

For peer review only

	6b	Explanation for choice of comparators – see last paragraph of introduction ✓
Objectives	7	Specific objectives or hypotheses – in "trial overview" ✓
Trial design	8	Description of trial design including type of trial (eg, parallel group, crossover, factorial, single group), allocation ratio, and framework (eg, superiority, equivalence, noninferiority, exploratory) – "trial overview" ✓
**Methods: Participants, interventions, and outcomes**		
Study setting	9	Description of study settings (eg, community clinic, academic hospital) and list of countries where data will be collected. Reference to where list of study sites can be obtained – "Participant identification" ✓
Eligibility criteria	10	Inclusion and exclusion criteria for participants. If applicable, eligibility criteria for study centres and individuals who will perform the interventions (eg, surgeons, psychotherapists) "Inclusion criteria" and "exclusion criteria". ✓
Interventions	11a	Interventions for each group with sufficient detail to allow replication, including how and when they will be administered – "trial procedures" ✓
	11b	Criteria for discontinuing or modifying allocated interventions for a given trial participant (eg, drug dose change in response to harms, participant request, or improving/worsening disease) "Treatment allocation and safety monitoring" ✓
	11c	Strategies to improve adherence to intervention protocols, and any procedures for monitoring adherence (eg, drug tablet return, laboratory tests) – "Treatment allocation and safety monitoring" ✓
	11d	Relevant concomitant care and interventions that are permitted or prohibited during the trial See "overview" and exclusion criteria. ✓
Outcomes	12	Primary, secondary, and other outcomes, including the specific measurement variable (eg, systolic blood pressure), analysis metric (eg, change from baseline, final value, time to event), method of aggregation (eg, median, proportion), and time point for each outcome. Explanation of the clinical relevance of chosen efficacy and harm outcomes is strongly recommended –"outcome measures" ✓
Participant timeline	13	Time schedule of enrolment, interventions (including any run-ins and washouts), assessments, and visits for participants. A schematic diagram is highly recommended (see Figure) Figure 1 and table 1 ✓
Sample size	14	Estimated number of participants needed to achieve study objectives and how it was determined, including clinical and statistical assumptions supporting any sample size calculations "sample size calculation" ✓

Recruitment	15	Strategies for achieving adequate participant enrolment to reach target sample size "participant identification" ✓

Methods: Assignment of interventions (for controlled trials)

Allocation:

Sequence generation	16a	Method of generating the allocation sequence (eg, computer-generated random numbers), and list of any factors for stratification. To reduce predictability of a random sequence, details of any planned restriction (eg, blocking) should be provided in a separate document that is unavailable to those who enrol participants or assign interventions IMP, treatment allocation and safety monitoring ✓
Allocation concealment mechanism	16b	Mechanism of implementing the allocation sequence (eg, central telephone; sequentially numbered, opaque, sealed envelopes), describing any steps to conceal the sequence until interventions are assigned Treatment allocation and safety monitoring ✓
Implementation	16c	Who will generate the allocation sequence, who will enrol participants, and who will assign participants to interventions Treatment allocation and safety monitoring ✓
Blinding (masking)	17a	Who will be blinded after assignment to interventions (eg, trial participants, care providers, outcome assessors, data analysts), and how Treatment allocation and safety monitoring ✓
	17b	If blinded, circumstances under which unblinding is permissible, and procedure for revealing a participant's allocated intervention during the trial Treatment allocation and safety monitoring ✓

Methods: Data collection, management, and analysis

Data collection methods	18a	Plans for assessment and collection of outcome, baseline, and other trial data, including any related processes to promote data quality (eg, duplicate measurements, training of assessors) and a description of study instruments (eg, questionnaires, laboratory tests) along with their reliability and validity, if known. Reference to where data collection forms can be found, if not in the protocol "trial procedures" ✓
	18b	Plans to promote participant retention and complete follow-up, including list of any outcome data to be collected for participants who discontinue or deviate from intervention protocols Treatment allocation and safety monitoring ✓
Data management	19	Plans for data entry, coding, security, and storage, including any related processes to promote data quality (eg, double data entry; range checks for data values). Reference to where details of data management procedures can be found, if not in the protocol Data analysis ✓

Statistical methods	20a	Statistical methods for analysing primary and secondary outcomes. Reference to where other details of the statistical analysis plan can be found, if not in the protocol – Data analysis ✓
	20b	Methods for any additional analyses (eg, subgroup and adjusted analyses) Data analysis ✓
	20c	Definition of analysis population relating to protocol non-adherence (eg, as randomised analysis), and any statistical methods to handle missing data (eg, multiple imputation) Data analysis ✓
**Methods: Monitoring**		
Data monitoring	21a	Composition of data monitoring committee (DMC); summary of its role and reporting structure; statement of whether it is independent from the sponsor and competing interests; and reference to where further details about its charter can be found, if not in the protocol. Alternatively, an explanation of why a DMC is not needed. Last paragraph of “Treatment allocation and safety monitoring” ✓
	21b	Description of any interim analyses and stopping guidelines, including who will have access to these interim results and make the final decision to terminate the trial Last paragraph of “Treatment allocation and safety monitoring” ✓
Harms	22	Plans for collecting, assessing, reporting, and managing solicited and spontaneously reported adverse events and other unintended effects of trial interventions or trial conduct- Treatment allocation and safety monitoring ✓
Auditing	23	Frequency and procedures for auditing trial conduct, if any, and whether the process will be independent from investigators and the sponsor “Role of the Sponsor”✓
**Ethics and dissemination**		
Research ethics approval	24	Plans for seeking research ethics committee/institutional review board (REC/IRB) approval – Ethics and dissemination ✓
Protocol amendments	25	Plans for communicating important protocol modifications (eg, changes to eligibility criteria, outcomes, analyses) to relevant parties (eg, investigators, REC/IRBs, trial participants, trial registries, journals, regulators) – “Trial monitoring and oversight” ✓
Consent or assent	26a	Who will obtain informed consent or assent from potential trial participants or authorised surrogates, and how (see Item 32) – Participant identification✓
	26b	Additional consent provisions for collection and use of participant data and biological specimens in ancillary studies, if applicable NA

Confidentiality	27	How personal information about potential and enrolled participants will be collected, shared, and maintained in order to protect confidentiality before, during, and after the trial – Section "data analysis"- ✓
Declaration of interests	28	Financial and other competing interests for principal investigators for the overall trial and each study site -Competing interests statement✓
Access to data	29	Statement of who will have access to the final trial dataset, and disclosure of contractual agreements that limit such access for investigators Section "data analysis"- ✓
Ancillary and post-trial care	30	Provisions, if any, for ancillary and post-trial care, and for compensation to those who suffer harm from trial participation – Last paragraph of "treatment allocation, blinding and safety monitoring" ✓
Dissemination policy	31a	Plans for investigators and sponsor to communicate trial results to participants, healthcare professionals, the public, and other relevant groups (eg, via publication, reporting in results databases, or other data sharing arrangements), including any publication restrictions Ethics and dissemination ✓
	31b	Authorship eligibility guidelines and any intended use of professional writers NA
	31c	Plans, if any, for granting public access to the full protocol, participant-level dataset, and statistical code NA
**Appendices**		
Informed consent materials	32	Model consent form and other related documentation given to participants and authorised surrogates
Biological specimens	33	Plans for collection, laboratory evaluation, and storage of biological specimens for genetic or molecular analysis in the current trial and for future use in ancillary studies, if applicable

*It is strongly recommended that this checklist be read in conjunction with the SPIRIT 2013 Explanation & Elaboration for important clarification on the items. Amendments to the protocol should be tracked and dated. The SPIRIT checklist is copyrighted by the SPIRIT Group under the Creative Commons "Attribution-NonCommercial-NoDerivs 3.0 Unported" license.

For peer review only
